# µLAS: Sizing of expanded trinucleotide repeats with femtomolar sensitivity in less than 5 minutes

**DOI:** 10.1038/s41598-018-36632-5

**Published:** 2019-01-10

**Authors:** Rémi Malbec, Bayan Chami, Lorène Aeschbach, Gustavo A. Ruiz Buendía, Marius Socol, Pierre Joseph, Thierry Leïchlé, Evgeniya Trofimenko, Aurélien Bancaud, Vincent Dion

**Affiliations:** 1 LAAS-CNRS, Université de Toulouse, CNRS, Toulouse, 31031 France; 2 Center for Integrative Genomics, Faculty of Biology and Medicine, University of Lausanne, Bâtiment Génopode, Lausanne, 1015 Switzerland; 3 Present Address: Department of Physiology, University of Lausanne, Rue du Bugnon 7, Lausanne, 1005 Switzerland

## Abstract

We present µLAS, a lab-on-chip system that concentrates, separates, and detects DNA fragments in a single module. µLAS speeds up DNA size analysis in minutes using femtomolar amounts of amplified DNA. Here we tested the relevance of µLAS for sizing expanded trinucleotide repeats, which cause over 20 different neurological and neuromuscular disorders. Because the length of trinucleotide repeats correlates with the severity of the diseases, it is crucial to be able to size repeat tract length accurately and efficiently. Expanded trinucleotide repeats are however genetically unstable and difficult to amplify. Thus, the amount of amplified material to work with is often limited, making its analysis labor-intensive. We report the detection of heterogeneous allele lengths in 8 samples from myotonic dystrophy type 1 and Huntington disease patients with up to 750 CAG/CTG repeats in five minutes or less. The high sensitivity of the method allowed us to minimize the number of amplification cycles and thus reduce amplification artefacts without compromising the detection of the expanded allele. These results suggest that µLAS can speed up routine molecular biology applications of repetitive sequences and may improve the molecular diagnostic of expanded repeat disorders.

## Introduction

Microfluidic technologies have a demonstrated potential for nucleic acids analysis, genetic testing, and sample preparation^1–4^. High efficiency separation of DNA, which is required for a wide variety of applications from sequencing to molecular diagnostics, has, for instance, been achieved by optimizing polymer matrices in capillary electrophoresis^5^. High sensitivity detection has been reported with a variety of on-line sample concentration strategies, including electrokinetic stacking with isotachophoresis or field amplification^6^, or by integrating a specific module in the workflow based on columns or magnetic particles^7^. We recently described a technology that simultaneously separates, enriches, and sizes DNA fragments within minutes^8^. It consists in monitoring DNA migration in a viscoelastic fluid using electro-hydrodynamic bi-directional actuation. The shearing of the liquid around the DNA molecules creates transverse viscoelastic forces, oriented towards the channel walls, which increase with the molecular weight (MW) of the DNA fragment and allow matrix-free separation. Furthermore, when operated in a channel with a funnel geometry, the technology forces similarly-sized fragments to accumulate at the same position in the funnel, thereby concentrating them^8^. However, because the first iteration of the device only had one channel, it could not accurately size DNA fragments. We thus designed a novel lab-on-chip system, termed µLAS (µLAboratory for DNA Separation), which is composed of two identical side-by-side channels operated with the same actuation parameters, so as to characterize a sample with respect to a reference DNA ladder in real time. To demonstrate its usefulness, we applied µLAS to the sizing of expanded trinucleotide repeats using minute amounts of amplified DNA, including samples derived from individuals with Huntington’s disease (HD) and myotonic dystrophy type 1 (DM1).

HD is caused by the expansion of a CAG/CTG repeat (henceforth we refer to repeat tracts using the non-transcribed strand in the sense orientation) in the first exon of the huntingtin gene (*HTT*)^9^. The resulting protein contains an abnormally long polyglutamine tract that renders it toxic, thereby precipitating neurodegeneration through an, as yet, unclear mechanism^10^. DM1 individuals, by contrast, harbor an expanded CTG repeat in the 3’ untranslated region on one allele of the dystrophia myotonica protein kinase (*DMPK*) gene^11–13^. The transcript produced is toxic and affects the processing of RNA transcripts in *trans*^14^.

Determining the precise size of the repeat tract is important because it determines in large part the severity of the disease for both HD and DM1^15,16^. For instance, normal *HTT* alleles have 26 or fewer CAG repeats, whereas HD-causing alleles have 36 or more. Intermediate *HTT* alleles with 27 to 35 CAG repeats are not believed to cause HD, but they are at risk of expanding into the pathological range in the next generation^17,18^. Similarly, DM1 individuals carry one normal allele with less than 37 CTGs and an expanded one of at least 50 CTGs; intermediate allele sizes carry risks for an expansion in the offspring^16,19^. Notably, repeat tracts are dynamic: they change in length at high frequencies in both germlines and somatic tissues. Repeat size determination is therefore critical to understand the causes of expansions and to identify molecular mechanisms for the deliberate contraction of repeat tracts to remove the cause of the disease^19^.

The main impediments in assaying repeat size is the lack of sensitivity and the labor-intensive nature of current assays^20^. Current diagnostic methods include the amplification of the repeat region from genomic DNA using a labelled primer followed by capillary electrophoresis^21^. In some instances, when the repeat tract is particularly large, a triplet-primed PCR is required in which one of the primers used anneals within the repeat tract^22^. This method has the advantage of being simple and thus most molecular biology laboratories have the necessary equipment. There are, however, significant disadvantages. For example, expanded CAG/CTG repeats are notoriously difficult to amplify and this can sometimes lead a falsely negative outcome in which the expanded allele is not detected. In addition, triplet-primed PCR does not work well when the expansion is interrupted, which can also lead to false negative^23^. Care is taken during these procedures, including the use of Southern blotting, to confirm any negative molecular diagnostics. This increases the time, labour, and cost of the tests.

For basic research applications, small-pool (SP-) PCR is the gold standard^24–26^. It consists of amplifying only a small number of alleles per PCR to bypass the preferential amplification of shorter alleles. This is followed by a Southern blot to visualize the PCR products. SP-PCR has the advantage of detecting single alleles and thus can determine both allele size and the length heterogeneity within a sample. It is unaffected (but also is blind to) the presence of interruptions. The method can take several days to perform and is labour-intensive. Given these shortfalls, there is a need for novel methods to determine repeat sizes that are sensitive, that provide information about repeat size variation, and that reduce the time and effort required to obtain the results.

Here we test the potential of µLAS for sizing of trinucleotide repeats associated with HD and DM1. We are particularly interested in evaluating whether the sensitivity of our assay is beneficial to the analysis of target alleles using small amounts of amplified DNA. For this, we performed a calibration of the technology for DNA fragments ranging from 100 to 4000 bp. We then established that heterogeneous populations of expanded alleles at the *HTT* and *DMPK* loci containing up to 750 CAGs can be detected in five minutes or less, thus covering the entire range of expansions seen in HD and much of that seen in DM1. We report that our assay allows us to decrease the amount of non-specific amplicons without affecting the accurate detection of the expanded allele. Therefore, µLAS speeds up the sizing of expanded trinucleotide repeats by reducing the number of cycles in pre-analytical steps and by drastically shortening the time required to size DNA. µLAS appears to be suitable for a wide array of molecular biology assays.

## Results

### System design and accuracy

**Figure 1:**
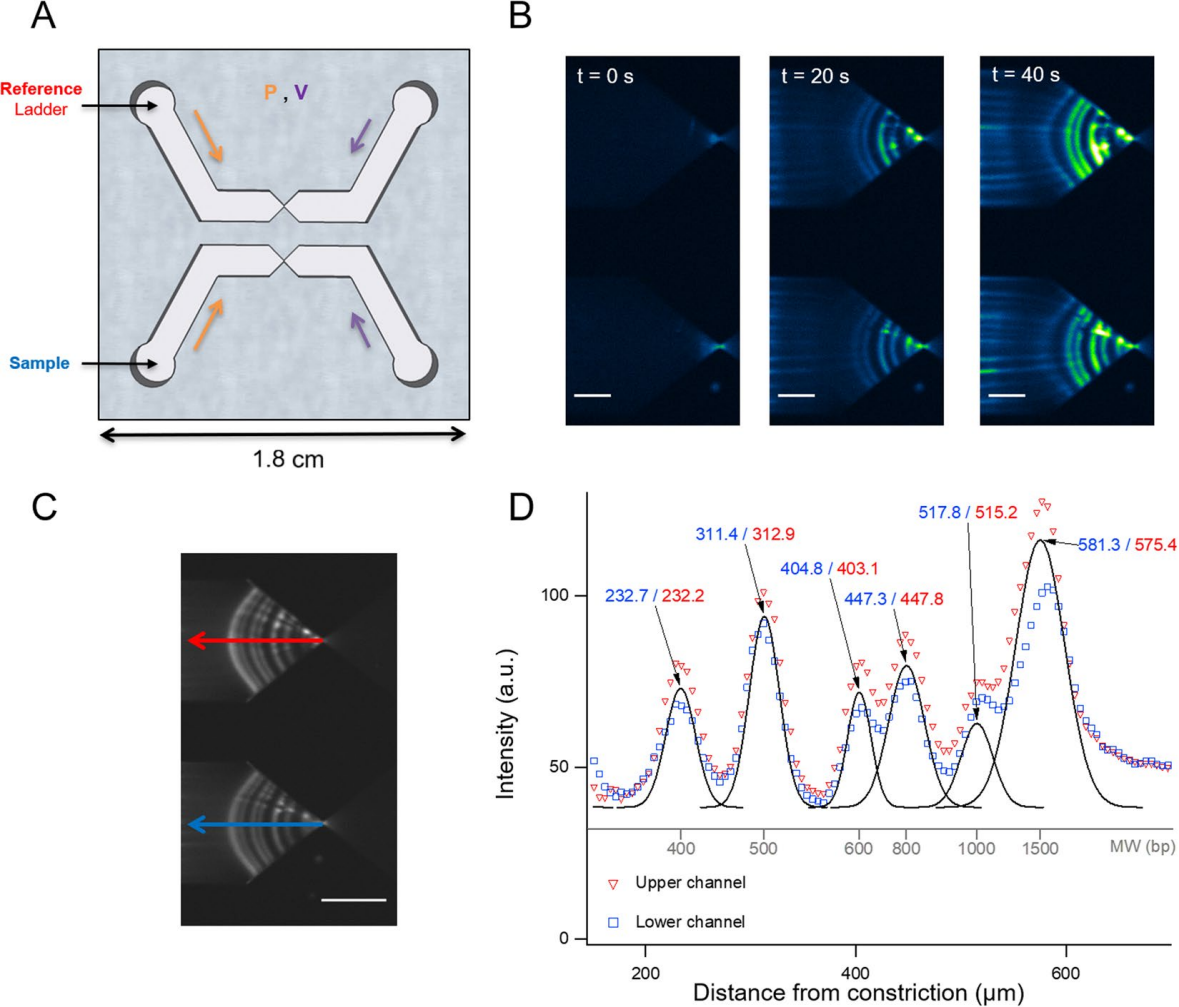
Functioning principle and accuracy of µLAS technology. (**A**) Sketch of µLAS chip with two independent channels actuated with the same pressure and voltage. Each channel contains a funnel region that functions to separate and concentrate DNA in line. (**B**) The time series of fluorescence micrographs show the two channels both containing a 100 bp ladder (diluted to 500 ng µL^−1^) being enriched after the electric field is applied at t = 0 s. After 40 s, 6 bands of the ladder are visible. Actuation parameters were set to 6 bar and 100 V. The scale bar corresponds to 200 µm. (**C**) An image is extracted from the time lapse shown in (**B**) after all the bands are concentrated and stabilized. The scale bar corresponds to 300 µm. (**D**) The graph shows the intensity distribution along the symmetry lines of each channel. Each peak is fitted with a Gaussian function to extract its position in µm, which is indicated with a tag. The peaks shown in inverted red triangles correspond to the upper channel in (**C**) while the blue squares correspond to the lower channel of the same figure.

When operating µLAS in a single channel system^8^, determining MW and concentration was time-consuming because the comparison between a fragment of interest and a MW marker had to be performed sequentially. We overcame this drawback by designing a new microfluidic chip composed of two channels, both operated with the same pressure and electric actuation parameters where the sample is injected in one channel and a reference ladder is introduced in the other for comparison as shown in the sketch of Fig. 1A. The functioning principle of µLAS is described in detail in^8^ and^27^. Briefly, DNA molecules are conveyed toward the constriction of the chip, and upon actuation of an electrophoretic force opposite to the hydrodynamic flow, they are stopped and enriched at positions dependent on their sizes. Figure 1B shows a time lapse of the concentration of the bands of a 100-bp ladder. Their distance to the constriction increases with DNA MW.

We tested the accuracy of µLAS for single band analysis by running the same ladder in the two channels (Fig. 1B,C) and by comparing the position of the 6 bands inferred from Gaussian fitting (Fig. 1D). The difference in position was typically less than 2 µm for fragments of 400 to 1000 bp (Supplementary Table S1). Given that the distance between the bands of 400 and 500 bp is 80 µm (Fig. 1D), the inaccuracy in positioning represents ~3 bp. The reproducibility of µLAS was then evaluated by running the separation and concentration process four times consecutively and by measuring the positions of five reference peaks in the range 0.5 to 3.3 kbp. We determined the standard deviation of less than 4 µm for these five peaks (Supplementary Table S1). Our estimates for the accuracy and reproducibility are therefore consistent, and they are smaller than the sizing accuracy (see below).

### µLAS for DNA sizing

**Figure 2:**
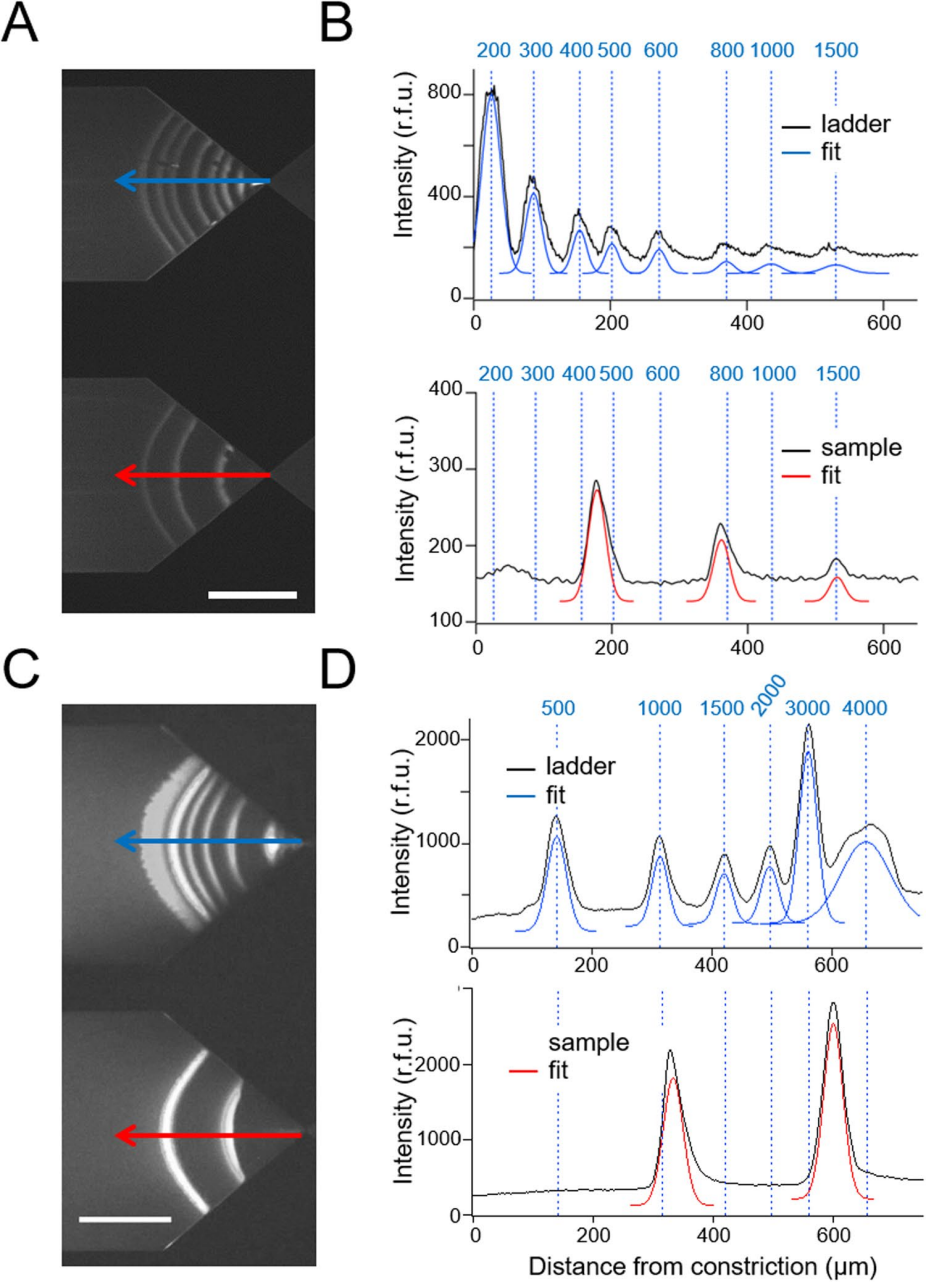
DNA separation and size identification. We used a reference signal with a DNA ladder in the upper channel and conveyed the analyte in the bottom channel. During calibration, we analyzed amplicons from non-repetitive sequences originating from plasmid DNA (45 cycles of PCR). (**A**) A fluorescence micrograph showing the two channels after concentration and separation during 30 s using a target sample with three fragments of 466, 798 and 1512 bp at 80 pg µL^−1^. Actuation parameters are set to 6 bar and 82 V, corresponding to maximal flow velocity and electric field of ~7.1 cm s^−1^ and 6.9 MV m^−1^, respectively. (**B**) The two plots represent the intensity profile along the two arrows represented in panel (**B**). The raw data is in black and the corresponding fits with Gaussian functions in blue and red. Based on the position of the center of each Gaussian peak in the ladder (top), we assign the size of the three bands in the sample by linear interpolation. More complex interpolation functions did not have a significant impact on accuracy of size determination. (**C**) The same experiment as in panel (**A**) with six bands of a kb ladder, and two bands of 1091 and 3314 bp diluted a concentration of 100 pg μL^−1^. Actuation parameters are set to 1 bar and 50 V. The results were obtained after 1 min of enrichment. (**D**) The two plots correspond to the fluorescence intensity distribution along the symmetry line of the two channels with the corresponding Gaussian fits. The scale bars correspond to 300 µm in (**A**) and 400 µm in (**C**).

We calibrated the sizing accuracy with the new chip design using three non-repetitive DNA fragments of 466, 798 and 1512 bp, which we produced by PCR amplification of plasmid DNA (see Methods). We fixed the DNA concentration within each band to 80 pg μL^−1^, which is about three orders of magnitude lower than the typical DNA concentration used for slab gel electrophoresis. We empirically defined the pressure and voltage to be able to visualize all 8 bands of the ladder simultaneously (Fig. 2A,B). The values of these parameters were 6 bar and 82 V, corresponding to a maximum flow velocity and an electric field of ~7.1 cm s^−1^ and 6.9 MV m^−1^, respectively (Supplementary Table S2). We recorded videos of DNA accumulation (Supplementary video 1), and systematically took micrographs after 30 s to 1 min for image processing. We extracted the intensity profile along the symmetry axis of the two funnels (blue and red arrows in Fig. 2A). To determine the size of the three target bands, we first used Gaussian fitting to measure the positions of all ladder bands as a function of their MW (Supplementary Fig. S1A), yielding an inhomogeneous power law scaling response over the size range 300 to 1500 bp. Hence, the position of the target bands was deduced by linear interpolation between the nearest ladder bands. We estimated the size of these three bands to be 456, 788 and 1550 bp, showing that the difference in DNA length between the readout and the nominal size was less than 3%, *i.e*., an accuracy of ~10 bp for a fragment of 350 bp, in this particular experiment.

We used the same procedure to calibrate higher MW DNA samples. The ladder had six bands from 0.5 to 4 kb, the sample was composed of two fragments of 1091 and 3314 bp, which we obtained by restriction of pUC19 hph (Fig. 2C,D). The flow and electric fields were reduced by factors of six and two, respectively, to resolve the six bands of the ladder. We determined the size of the two target bands to be 1089 and 3417 bp, and concluded that the difference between the readout and the nominal size was also 3% within a range of 100 bp to 4 kb. Notably, the analysis of the ladder allowed us to compute the resolution length, as defined by the size difference between two bands divided by the separation resolution^28^. The resolution length corresponds to the minimal size between two baseline-resolved peaks separated in one single channel^29^. We found that this value varied between 40 and 70 bp in the range of 200 to 1000 bp (Supplementary Fig. S1B), implying that sizing experiments could not be carried out by mixing the ladder and the sample in a single channel due to risks of overlap. This justified the use of a dual channel chip to achieve a precision of 3%.

**Figure 3:**
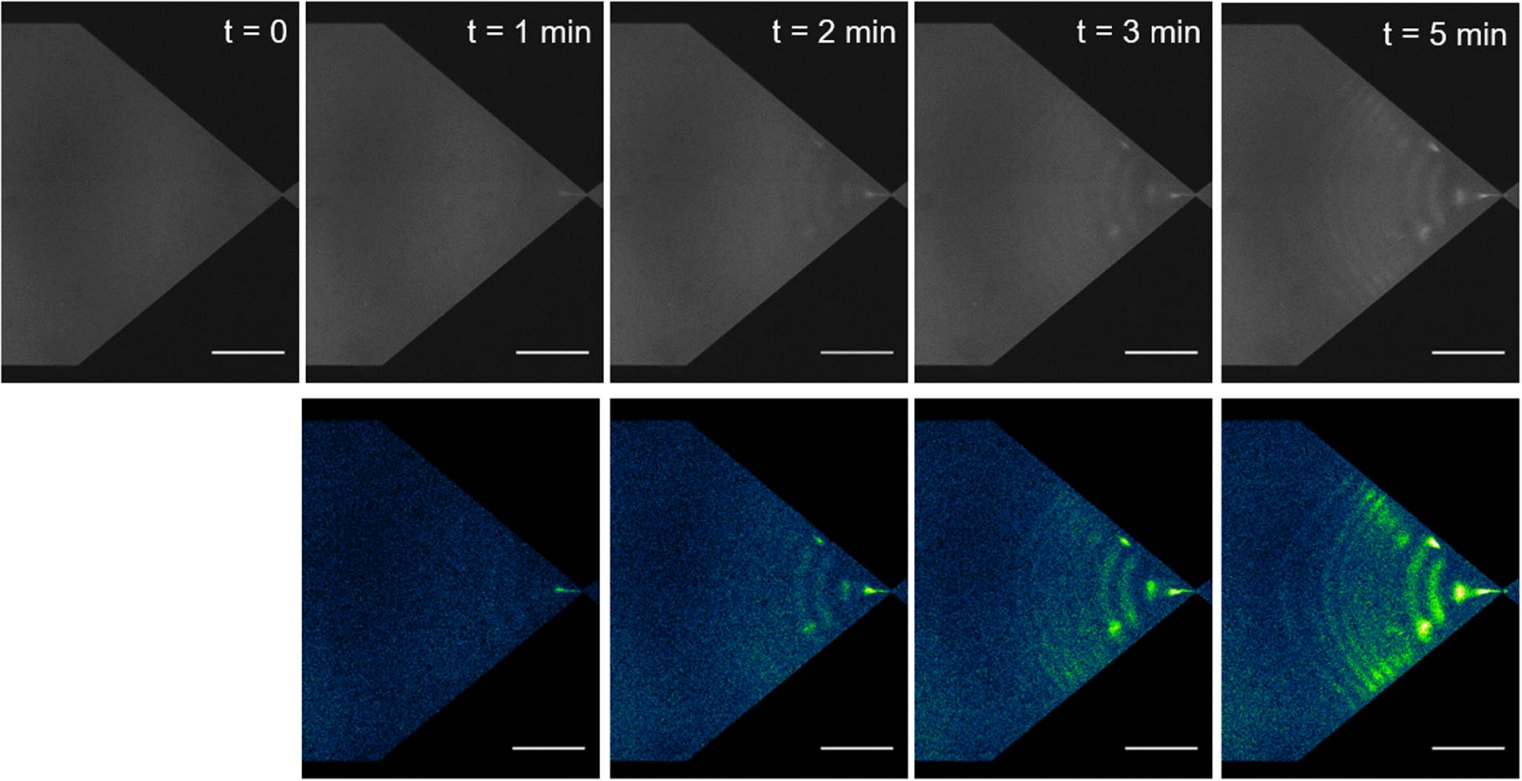
Detection of DNA ladder fragments at 100 fg µL^−1^. The time series in the upper panel shows fluorescence intensity at the constriction using a 100 bp DNA ladder diluted at 1 pg µL^−1^. Actuation parameters are the same as in Fig. 2A. Taking the fluorescence micrograph at t = 0 s as a reference, the lower panel represents background subtracted intensity profiles, in which the presence of the 8 bands of the ladder appear after five minutes (see Fig. 2A,B). The scale bars correspond to 300 µm.

Finally, we evaluated the lower limit of detectable DNA concentration after five minutes of concentrating and separating fragments with the 100 bp ladder at a total concentration of 1 pg µL^−1^, or ~100 fg µL^−1^ per band. We observed a build-up in intensity for all bands of the ladder within five minutes (Fig. 3). Notably, given that the flow rate was 0.09 µL min^−1^ (Supplementary Table S2), our analysis after five minutes was carried out with a volume of ~0.5 µL, and each band contained ~50 fg or equivalently ~50,000 molecules of 1000 bp. The Fragment Analyzer, a high-sensitivity equipment for nucleic acid detection, has a lower limit of detection of about ~5 pg µL^−1^ with a processed volume of 20 µL, according to the manufacturer. The sensitivity of µLAS was therefore 50-fold and 2,000-fold greater in concentration and mass, respectively. In comparison to other microfluidic technologies, we reach a limit of detection that is ten times greater than that reported for rheotaxis^30^ or electrokinetic stacking^31^. Nevertheless, these technologies are adequate to process fragments larger than 2 kb or smaller than 100 bp, respectively. Furthermore, we could determine the accuracy of the concentration measurements of each DNA fragment in Fig. 2A by comparing the total intensity of each band to that of their nearest neighbor in the ladder. We found that the error on the concentration measurements was 30% (Supplementary Fig. S2).

Altogether, from the moment the samples were loaded in the channels and the hydrodynamic and voltage actuations were turned on, µLAS allowed us to estimate DNA MW and concentration within five minutes with an accuracy of 97% and 70%, respectively, for fragments between 100 bp and 4000 bp and with a sensitivity of ~100 fg µL^−1^.

### Using µLAS to measure the size of expanded CAG repeats

**Figure 4:**
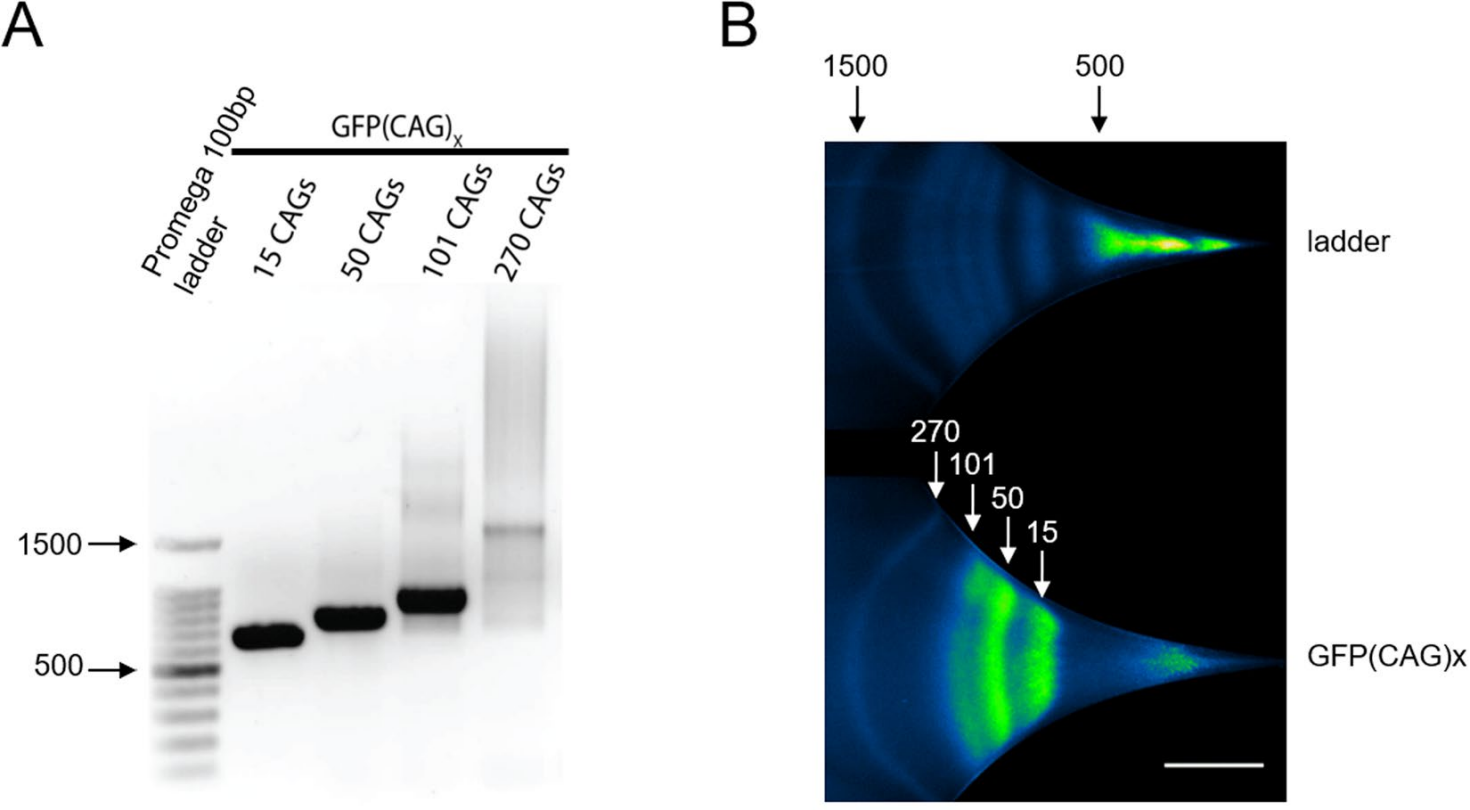
Measuring CAG repeat expansions in GFP(CAG)_x_ cell lines. (**A**) Four samples containing 15 to 270 CAGs after 35 PCR cycles run on a 1% TAE agarose gel. The samples are pools of three reactions. (**B**) The four samples amplified using 25 PCR cycles were mixed together and processed during 30 s in our microfluidic platform. The scale bar corresponds to 300 µm.

Our results prompted us to apply µLAS to situations in which determining precise fragment sizes using minute amounts of DNA is relevant. Thus, to test whether we could size expanded CAG-containing sequences, we amplified a repeat tract from a single copy GFP transgene integrated in HEK293-derived cell lines (GFP(CAG)x, where x is the number of repeats harbored by the transgene^32^. We obtained amplicons from GFP(CAG)x cells carrying 15, 50, 101, and 270 CAGs, as determined by Sanger sequencing, with expected amplicon sizes of 672, 777, 930 and 1437 bp, respectively. We limited the PCR amplification to 25 cycles to reduce the possibility of non-specific amplification (see below). Analysis of the reaction product with a Fragment Analyzer showed that longer repeat sizes were more heterogeneous, as expected (Supplementary Fig. S3A). We estimated the fragment sizes to be 675, 782, 920 and 1410 bp, implying that the error in sizing was 0.4, 0.6, 1.1, and 1.9%, respectively (Fig. 4B). In all cases, therefore, we obtained bands of the expected sizes in one single analysis with the four samples pooled together using conditions that allow each band to be well resolved. Notably, to obtain enough material to be able to visualize the fragment with 270 CAGs by agarose gel electrophoresis, we had to pool the product of three PCRs (Fig. 4A), which emphasizes both the difficulty in obtaining large quantities of DNA from these sequences and the enhanced sensitivity of µLAS. Thus, µLAS accurately determined the CAG repeat size in all four samples.

### Characterization of PCR products of the *HTT* locus

**Figure 5:**
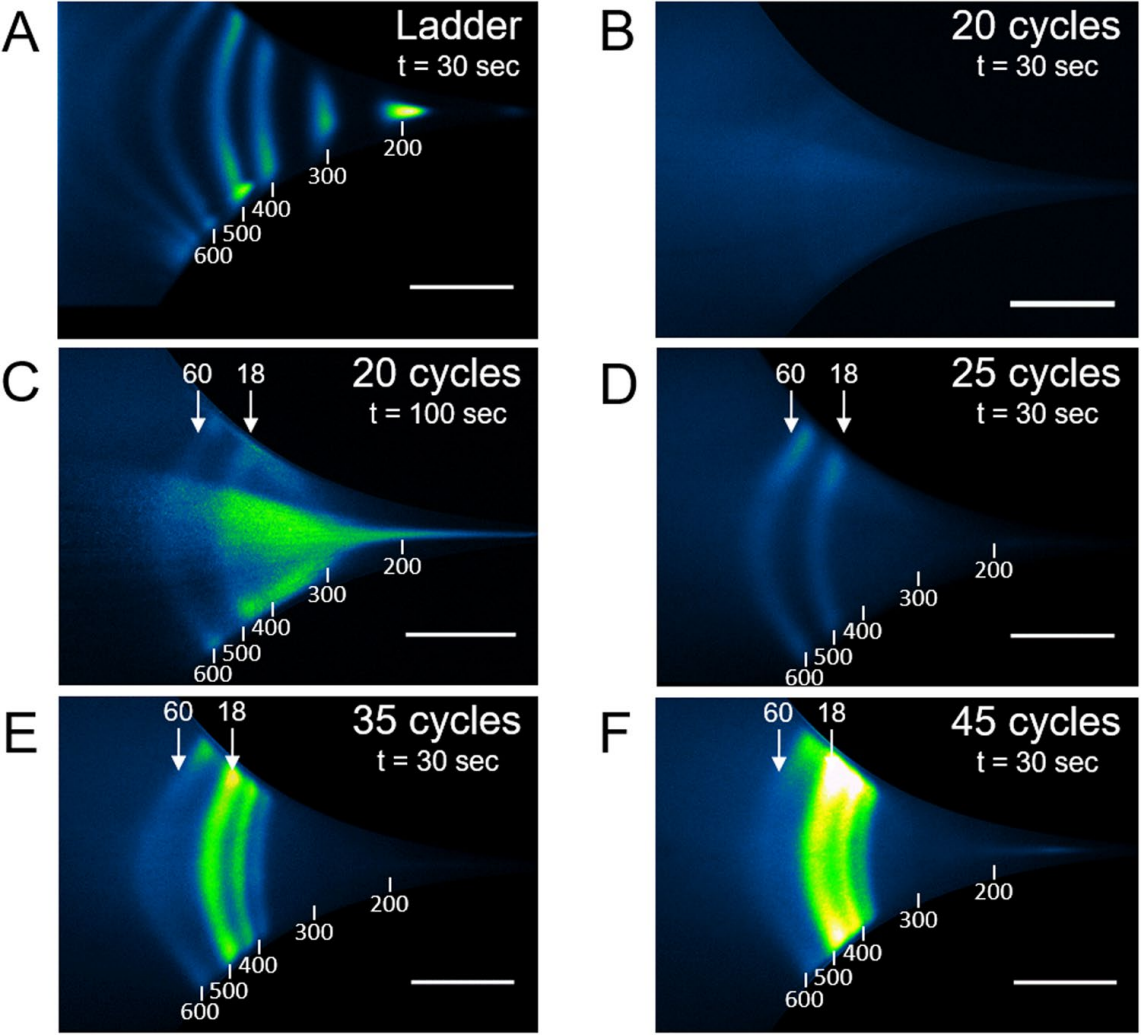
Analysis of PCR amplification from genomic DNA of a patient with 18 and 60 CAG repeats. Amplicons obtained after 20, 25, 35, and 45 PCR cycles are conveyed in the microfluidic channel using a constriction with a parabolic profile and actuation parameters of 2 bar and 130 V, corresponding to ~5.7 cm s^−1^ and 1.52 MV m^−1^ for the maximum flow velocity and electric field, respectively. Each panel shows the fluorescence micrographs. The corresponding intensity profiles along the symmetry line of the channel are shown in Supplementary Fig. S4. (**A**) 100 bp MW ladder after 30 s of concentration. (**B**) 20 cycles of PCR after 30 s of concentration. (**C**) 20 cycles of PCR after 100 s of concentration. (**D**) 25 cycles of PCR after 30 s of concentration. (**E**) 35 cycles of PCR after 30 s of concentration. (**F**) 45 cycles of PCR after 30 s of concentration. The vertical blue lines in the plots in Supplementary Fig. S4 are the positions of the bands of the ladder, as inferred from Gaussian fitting of the data in (Supplementary Fig. S4A). The scale bars correspond to 200 µm.

**Figure 6:**
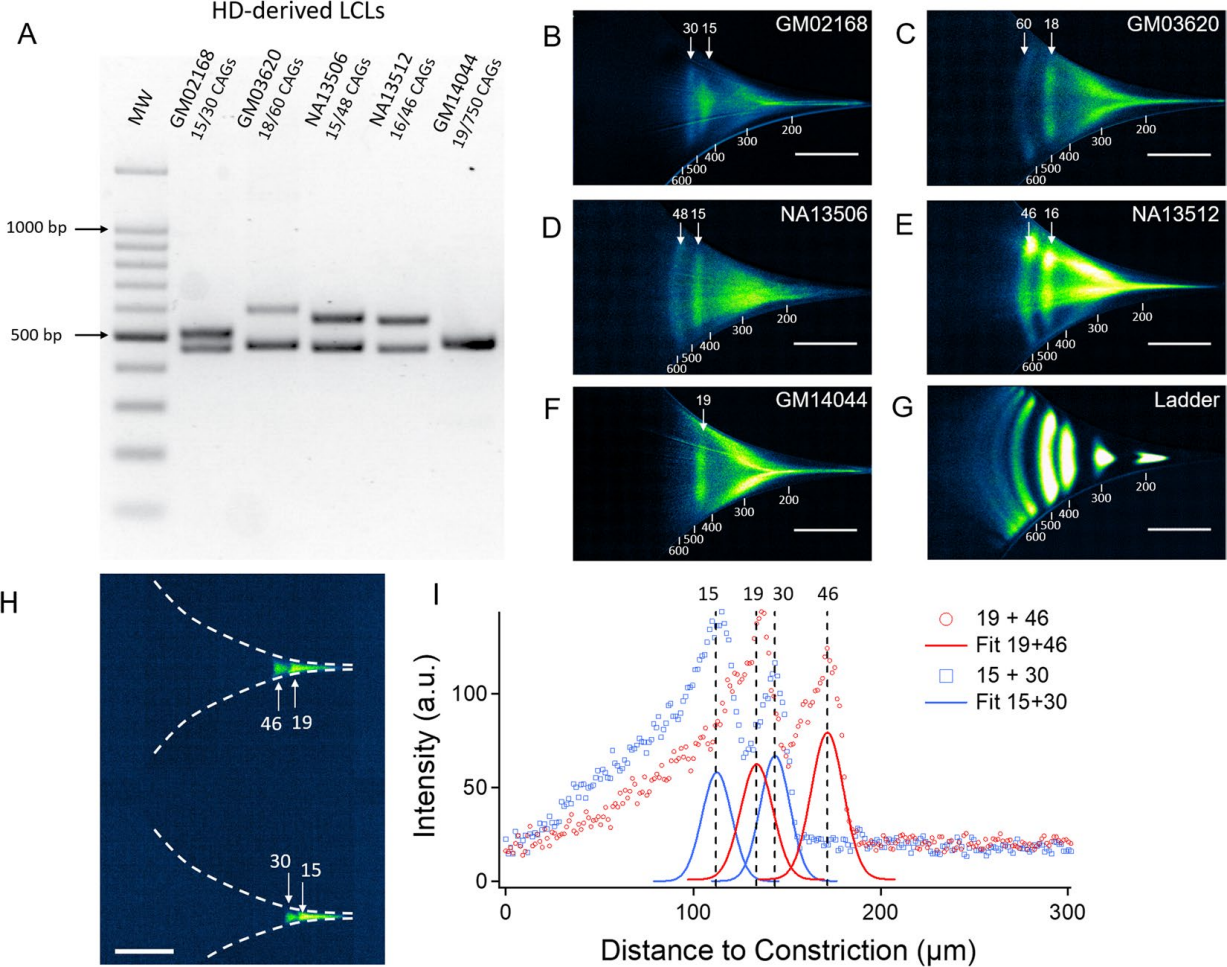
Analysis of HD patient-derived samples after 25 cycles of PCR. (**A**) 1% TAE agarose gel electrophoresis of patient-derived amplicons (45 cycles, 500 ng loaded). (**B–G**) The fluorescence micrographs represent the PCR products conveyed in the chip at 2 bar and 160 V, i.e. maximum hydrodynamic and electric fields of ~5.7 cm s^−1^ and 1.9 MV m^−1^, respectively, and enriched during 30 s. The plots obtained from the intensity distribution along the channel symmetry axis are shown in Supplementary Fig. S6. The ladder concentration was set to 15 pg µL^−1^ (panel (G)). (**H–I**) Analysis of four alleles with 15, 19, 30, and 46 repeats after their excision and purification from agarose gels. The micrograph shows the experiment and the plot is the corresponding intensity distribution along the channel symmetry axis. The scale bars correspond to 300 (**B–G**) or 200 µm (**H**). The results in (**H**) were obtained after enrichment during 45 s.

To determine whether we could apply µLAS to more clinically relevant complex samples, we next monitored the repeat size at the *HTT* gene in genomic DNA isolated from a lymphoblastoid cell line (LCL) derived from a HD patient (GM03620). This individual bore one allele with 18 CAGs and another with 60 repeats, according to our Sanger sequencing. Using primers flanking the repeat tract (and including the polymorphic CGG repeat abutting the CAG repeat^22^), we expected the amplification of fragments measuring 455 and 581 bp, respectively. To test for the sensitivity of the assay, we sized the repeat tracts in amplicons after 20, 25, 35, and 45 cycles of amplification, for which the total DNA concentration was 3.5, 5.7, 72, and 104 ng µL^−1^, respectively, as inferred from absorbance spectroscopy. The concentration of each sample was then set to 35 pg µL^−1^ for µLAS analysis, meaning that they were diluted at least 100-fold and the total concentration of the ladder was set to 400 pg µL^−1^, or ~40 pg µL^−1^ per band. This concentration range allowed us to characterize each sample within one minute or less (Fig. 5A–F). After 25 cycles of PCR, two bands of 478 and 584 bp were consistently detected (Table 1, Fig. 5D), corresponding with the expected PCR products. Note that the analysis of the sample after 20 cycles of amplification required a longer time of concentration of 100 s because the signal was undetectable after 30 s (Fig. 5B). In addition, the two bands were fainter due to the presence of a smeary pattern (Fig. 5C), yet the measurement of their size of 485 and 595 bp was qualitatively comparable to that after 25 cycles (Table 1). After 35 cycles of PCR, two additional bands of 397 and 434 bp were observed (Fig. 5E and see intensity profile in Supplementary Fig. S4D). The pattern appeared to be qualitatively comparable after 45 cycles of amplification, although the allele with 60 repeats became much dimer compared to the low MW bands (Fig. 5F). Notably, an additional 735 bp band 20-fold more diluted appeared after 45 rounds of amplification (Supplementary Fig. S5). In contrast, sample analysis by agarose gel electrophoresis after 45 cycles only revealed two bands at ~480 and 610 bp (Fig. 6A, sample GM03620), probably because of insufficient sensitivity and/or the inability to resolve bands of similar MW. Nevertheless, we confirmed the presence of multiple peaks after 45 cycles using the Fragment Analyzer, and that of two peaks at 25 cycles (Supplementary Fig. S3B). These extra bands may be non-specific by-products or heteroduplexes with hybrids of the two alleles.

**Table 1:** Sizes and concentrations of GM03620 HTT PCR products.

# of cycles	20		25		35				45				
Amplicon	Allele 1	Allele 2	Allele 1	Allele 2	Unspe 1	Unspe 2	Allele 1	Allele 2	Unspe 1	Unspe 2	Allele 1	Allele 2	Unspe 3
Fragment size (bp)	485 +/− 15	595 +/− 18	478 +/− 14	584 +/− 18	397 +/− 12	434 +/− 13	485 +/− 15	596 +/− 18	399 +/− 12	430 +/− 13	480 +/− 14	575 +/− 17	735 +/− 22
Concentration (ng/µL)	0.1 +/− 0.02	0.06 +/− 0.01	3.2 +/− 0.5	5.0 +/− 1.5	38 +/− 6	117 +/− 18	251 +/− 38	136 +/− 20	117 +/− 18	338 +/− 51	506 +/− 76	233 +/− 35	5.4 +/− 3
Enrichment factor	—	—	32	83	—	—	65	21	3.4	2.8	2.5	1.9	—

The position of the allelic and nonspecific bands in the sample and their concentration before dilution for on-chip analysis are deduced from the data presented in Fig. 5. The high MW band only detected after 45 cycles is deduced from the fluorescence micrograph shown in Supplementary Fig. S5. The last row presents the enrichment factor inferred from the concentration ratio between consecutive cycles of the PCR amplification.

We also assessed the amplification factor for each band by measuring the increase in the concentration of the amplicons with the number of cycles. We calculated this factor to be maximal between 20 and 25 cycles for the HD alleles with about two copies of DNA produced per cycle (last row in Table 1). In contrast, we observed a decrease in the amplification efficiency down to 1.05 copies per cycle between 35 and 45 cycles, probably due to the competitive formation of the extra bands as well as the consumption of the primers and dNTPs. From these results, we concluded that to maintain high levels of accuracy and eliminate the production of amplification artefacts, the determination of the repeat size of the *HTT* locus should be carried out with 25 cycles of amplification and with single band concentrations of ~400 pg µL^−1^.

### Analysis of patient-derived samples

Next we asked how widely our method is applicable by performing more detailed analyses on patient-derived samples. Specifically, we determined CAG repeat lengths in three more HD patient samples and an unaffected individual, covering the whole range of repeat size seen in HD. The shorter alleles contained 15 to 19 repeats, corresponding to amplicons in the range of 446 to 458 bp, whereas the larger alleles presented 30, 46, 48, 60 and 750 repeats (Table 2). These corresponded to size differences between the two alleles of 45, 84, 99, 126 and 2193 bp, respectively. The samples were collected after 25 cycles of PCR at a typical concentration of ~1 ng µL^−1^. They were diluted by a factor of ~100 into the separation buffer, and a 100 bp ladder was prepared at 15 pg µL^−1^. They were processed on chip during 30 s (Fig. 6B–G and corresponding fluorescence intensity linear profiles in Supplementary Fig. S6). For four of the five samples, we could detect two alleles with lengths consistent with the prediction based on Sanger sequencing and agarose gel electrophoresis after 45 cycles of amplification (Table 2 and Fig. 6A). For instance, the distance between the two alleles in sample NA13506 (15 and 48 CAGs, 446 bp and 545 bp) and NA13512 (16 and 46 CAGs, 449 bp and 539 bp) appeared to be comparable (panels D-E in Fig. 6), as expected from the similar size difference of 33 and 30 CAG repeats, respectively. Furthermore, the physical distance between the bands in sample GM02168 (15 and 30 repeats, 446 bp and 491 bp), which corresponds to 15 repeats, was smaller, and that of sample GM03620 (18 and 60 CAGs, 455 bp and 581 bp) with 42 repeats was slightly greater (panels B-C in Fig. 6). The concentration of these fragments of 400 to 600 bp was in the range of 50 to 600 pg µL^−1^, in agreement with our measurements at different stages of the PCR amplification (Table 2).

**Table 2:** Sizes and concentrations of HD and DM1 patient samples after 25 cycles of PCR.

Sample	HD										DM1					
	NA13506		NA13512		GM03620		GM14044		GM02168		GM04604	NA05164			GM03756A	
Allele size (bp)	446	545	449	539	455	581	458	2193	446	491	1051	1099	2167		1051	2386
Measured size (bp)	443 +/−13	544 +/−16	473 +/−14	562 +/−17	477 +/−14	590 +/−18	477 +/−14	1850 +/−55	455 +/−14	513 +/−16	1052 +/−32	1144 +/−34	1714 +/−34	2816 +/−34	1100 +/−100	1800 +/−100
Expected number of CAG/CTG repeats	15	48	16	46	18	60	19	750	15	30	5	21	377		5	450
Measured number of CAG/CTG repeats	14 +/− 4	48 +/− 5	24 +/− 5	54 +/− 6	25 +/− 5	63 +/− 6	25 +/− 5	636 +/− 18	18 +/− 5	38 +/− 5	5	31	221	594	21	250
Measured concentration (ng/µL)	0.05	0.05	0.2	0.3	0.4	0.2	0.5	0.2	0.1	0.2	0.5	0.4	0.1	0.03	ND	ND

**Figure 7:**
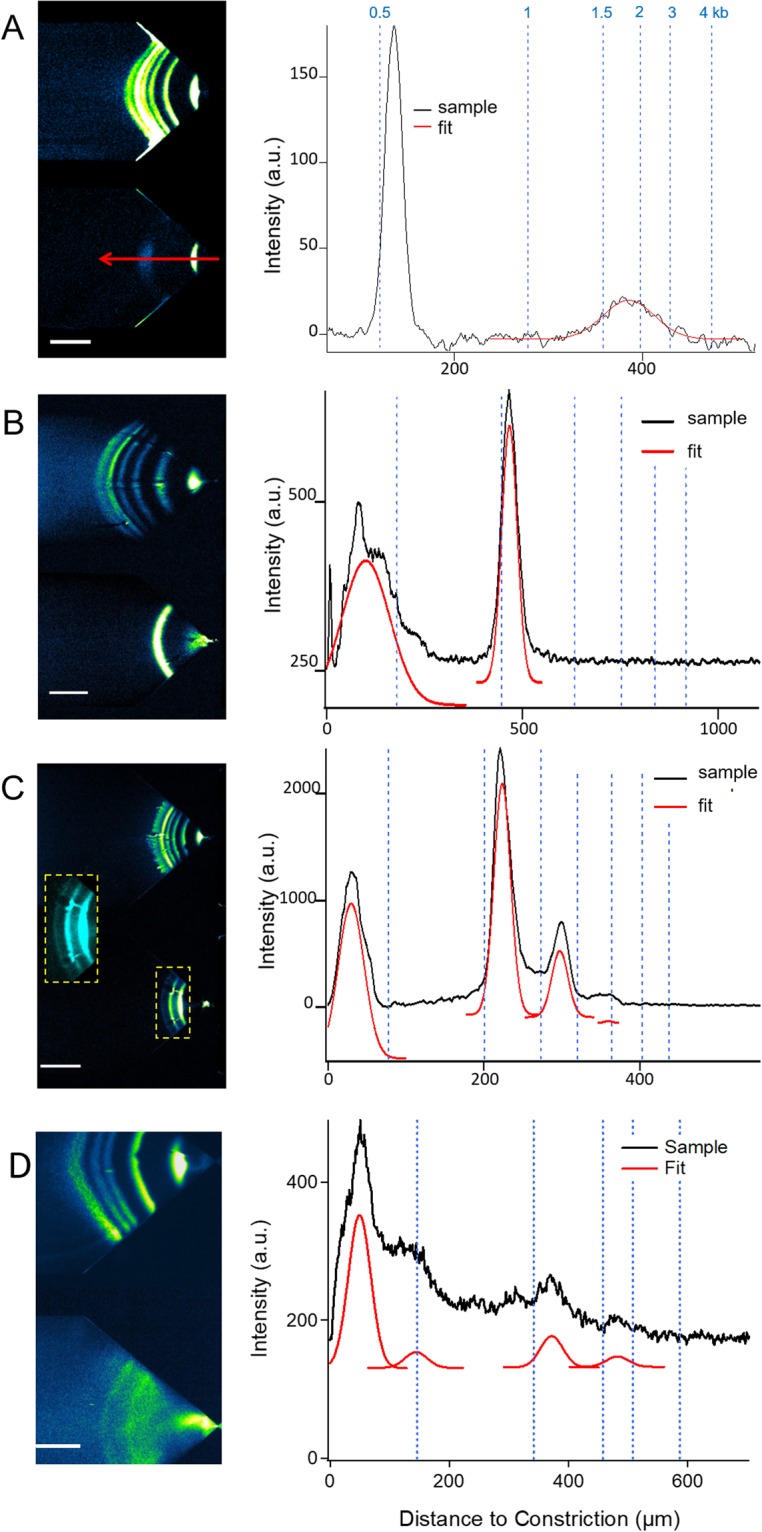
Analysis of GM14044 HD sample and DM1 samples with 1 kb ladder reference. (**A**) The fluorescence micrograph of the GM14044 PCR products conveyed in the chip at 1 bar and 66 V, i.e. maximum hydrodynamic and electric fields of ~1.2 cm s^−1^ and 5.5 MV m^−1^, respectively, and enriched during 30 s. The corresponding intensity profile along the symmetry axis of the channel is plotted on the right. Note that the micrograph with a lower tension of 20 V, i.e., with a brighter high MW band but a poorer size resolution, is shown in Supplementary Fig. S6G. (**B**) Analysis of the *DMPK* locus of an unaffected individual (GM04604) with the µLAS fluorescence micrograph and the corresponding intensity profile. Actuation parameters are ~1 cm s^−1^ and 4.7 MV m^−1^. (**C,D**) Same as in (**B**) with the DM1-derived samples (NA05164 and GM03756A) with actuation parameters of ~1 cm s^−1^ and 3.1 MV m^−1^ or 3.5 MV m^−1^, respectively. The scale bars correspond to 300 or 200 µm in panels A-C or D, respectively. The area underlined with a dashed line is zoomed in by a factor of 167% in the inset. The bands below 500 bp in panels B-D are likely to be primers that were not removed in these experiments. The results in B-D were obtained after enrichment of 30 s to 1 min.

The size of the bands, the corresponding number of CAG/CTG repeats, and their concentration was calculated from the data presented in Figs 6 and 7.

Notably, we could separate the two alleles separated by 15 repeats in sample GM02168 (15 and 30 CAGs, 446 bp and 491 bp). Yet, the genomic distance of 45 bp was comparable to the resolution length of our technology of ~50 bp (Supplementary Fig. S1B). Accordingly, as we purified and pooled the alleles of 15, 19, 30, and 46 repeats after gel electrophoresis, we observed that the four bands could not be separated in a single channel due to the overlap between the alleles with 19 and 30 repeats (Supplementary Fig. S8). The four alleles could nevertheless be sized in a dual channel chip (Fig. 6H,I), showing that µLAS can separate two alleles with more than 15 CAG repeats, and measure their lengths with a precision of plus or minus four repeats (Table 2).

For sample GM14044, which contains one allele with 19 CAGs and a longer and more heterogeneous expanded allele averaging ~750 CAG repeats (Supplementary Fig. S7), we could only detect the shorter allele using these initial conditions (Fig. 6F). We therefore switched to higher MW analysis with the 1 kb ladder as reference, and detected two bands of ~500 and 1850 bp (Fig. 7A). The size of the high MW band is smaller than the expected length of 2193 bp. By contrast, we used the only other method to detect such small amounts of DNA, Fragment Analyzer, and found a very faint band with a size of 1120 bp (Supplementary Fig. S3). This was expected because expanded CAG/CTG repeats run anomalously fast through acrylamide-based matrices^33^, like the one used by the Fragment Analyzer. Although µLAS appears to underestimate the allele length for very long repeats, we note that the width of this high MW band was 2.5 times larger than that of the 2 kb fragment of the ladder, indicating the occurrence of genomic instability for this expanded allele, as seen with the SP-PCR (supplementary Fig. S7). We conclude that µLAS allows the detection of expanded repeat tracts covering the whole range of sizes seen in HD.

Furthermore, we applied our technology to a DM1-derived sample. A single band of 1052 bp was detected for the sample from an unaffected individual (GM04604; Fig. 7B, Table 2), in good agreement with the expected size of 1051 bp for two alleles of 5 CTGs. We then processed two samples with expanded allele from the DM1 individual (NA05164 and GM03756A), which harbor expanded alleles determined to be 377 and 450 CTG repeats by conventional diagnostic methods, respectively^34^. Surprisingly, SP-PCR analysis revealed that these samples contained a large number of contractions in addition to some alleles corresponding to the expected size (Supplementary Fig. S7). Consistently, a 30 s analysis with µLAS revealed four bands for NA05164 with the normal allele of 1144 bp, and two higher MW bands of 1714 and 2816 bp, i.e., corresponding to 221 and 594 CTG repeats, respectively (Fig. 7C, note the inset for the high MW band). The longer band, however, is broad, covering a range between 2 and 3 kbp. The fitting of the signal appears to overestimate the size of the repeat tract compared to the SP-PCR data (Supplementary Fig. S7). The concentration of this larger band was four- and twelve-times lower in comparison to that of 1714 and 1144 bp, respectively, perhaps leading to the inaccuracy in determining the precise repeat size. Furthermore, the bands in the GM03756A sample were poorly resolved (Fig. 7D) due to the presence of a smear, that likely reflected the degree of instability of this allele seen in SP-PCR (Supplementary Fig. S7). Nevertheless, a normal and an expanded allele of 1100 and 1800 bp, respectively, could be detected. Although the MW of the expanded allele was significantly less than the expected value of 2400 bp, we could not detect any band larger than 2000 bp even after more concentration in µLAS (not shown). Furthermore, bands larger than 2000 bp were infrequent according to our SP-PCR analysis. Altogether, these results suggest that µLAS is applicable to a wide variety of samples and allele-length heterogeneity contents.

## Discussion

In this study, we designed a new microfluidic system that enables the separation, concentration, and sizing of DNA fragments within minutes using miniscule amounts of material. We expect this to be advantageous for routine molecular biology work, as it would save time usually spent during agarose gel electrophoresis. Moreover, we show that because of the high sensitivity of the assay, we could use shorter PCR programs and reduce the risk of amplification artifacts associated to non-specific by-products or heteroduplexes of molecules with different size repeats. We further demonstrate that µLAS can detect expanded repeats and detect heterogeneous alleles from patient-derived samples with an average repeat size of up to ~750 CAGs. Our results thus cover the entire range of expansions seen in HD. Importantly; the results obtained from the DM1 samples appear to reflect the instability seen by SP-PCR. Therefore, µLAS could facilitate molecular biology applications involving repetitive and unstable sequences. This is highlighted by the analysis of GM14044 by gel electrophoresis, in which only the short allele is detected (Fig. 6A). The lower concentration of the expanded allele is likely due to its instability as well as to the lower amplification efficiency. With its sensitivity, µLAS is particularly well suited to detect variability in repeat size within the same population. It further provides a quantitative size profile in a few minutes as opposed to SP-PCR, which takes several days. Yet, there may be some challenges with measuring very large alleles. For example, the buffer conditions described here poorly resolve fragment sizes above 4 kbp. This has significant impact for samples from individuals with congenital DM1, for example, in which the repeat tract can be beyond 4 kb in size, but the viscoelastic matrix can be optimized to allow the separation of larger DNA fragments by, for example, reducing the PVP viscosity 15-fold (not shown). Nevertheless, µLAS would be particularly useful for working with samples from most patients with expanded repeat disorders as well as most mouse models. We suggest that the sensitivity, speed, and the added instability data provided by µLAS may find applications in molecular diagnostics of expanded repeat disorders. Thorough testing of µLAS for such an application is, however, beyond the current study.

Given the sensitivity of µLAS, we suggest that non-PCR-based approaches may be compatible with our microfluidic method. For example, it may be possible to use *in vitro* CRISPR-Cas9 digestion^35^ of genomic DNA to release a single DNA fragment corresponding to a locus of interest and then separate, concentrate, and size it without the need for PCR amplification. Given our limit of detection of 50,000 gene copies and assuming high-efficiency Cas9 digestion, we could perform our analysis with one million cells in an analytical volume of 20 µL, which would be processed with a high analytical flow rate of 1–10 µL/min that is readily accessible with channels of 5 µm in height (not shown). Because this approach does not rely on PCR amplification with serial cycles but only on the sensitivity of µLAS, artifacts related to DNA synthesis would no longer be a problem. Therefore, this approach, which obviously requires minimal off-target DNA restriction by Cas9, would perform the equivalent of a Southern blot but on a microfluidic chip, within minutes.

## Materials and Methods

### Chemicals and reagents

Chemical reagents were purchased from Sigma-Aldrich, the 100 bp and 1 kb ladder from Ozyme (note that the 100 bp ladder contained 9 bands in 2015 (Fig. 1), and 11 bands since 2016), and fluorophores from Thermofisher. The plasmid pUC19-hph was provided by O. Gadal^36^. The electrophoretic buffer for µLAS analysis was composed of 1X Tris-Borate-EDTA (TBE, 89 mM Tris, 89 mM boric acid, 2 mM EDTA) supplemented with 1.3 MDa Polyvinylpyrrolidone (PVP) and Dithiothreitol (DTT) dissolved at 5% and 2% in weight, respectively, and YOPRO-1 at 100 nM for DNA fluorescent labelling. We inferred the viscosity and elastic relaxation time of the solution to be 35 mPa.s and 30 ms, respectively^27^. Solutions were filtered with 0.22 µm filters. Samples and ladders were diluted into the electrophoretic buffer at typical concentrations of ~100 pg µL^−1^.

### Cell lines and genomic DNA extraction

Patient-derived LCLs and DNA samples were obtained from the Coriell biorepository (coriell.org – Table 2). LCLs were grown in RPMI supplemented with 15% fetal bovine serum, 2 mM L-GlutaMAX, and penicillin/streptomycin. GFP(CAG)x cells were grown in DMEM containing 10% fetal bovine serum and penicillin/streptomycin as in^32^. Genomic DNA was extracted using the NucleoSpin Tissue kit from Macherey-Nagel according to the manufacturer’s protocol.

### PCR amplification and DNA templates

The PCR amplification for the HD and GFP(CAG)x samples was done with five units of MangoTaq DNA polymerase (Bioline) using 100 ng of genomic DNA (equivalently 30,000 allele copies) in the supplied buffer supplemented with 1 mM MgCl_2_, 200 µM dNTP, 0.5 µM of each primer, and 3% DMSO. The DM1 samples were amplified with four units of MyFi Taq (Bioline) with the buffer provided and the same primer concentrations. The PCR program started with an initial step for five min at 95 °C followed by five cycles of 20 s at 95 °C, 20 s at 52 °C, and 60 s at 72 °C. The following 15 to 40 cycles were 30 s at 95 °C, 30 s at 55 °C, and 90 s at 72 °C. A final step of 10 minutes at 72 °C was performed. The primers and DNA templates used in this study are found in Supplementary Table S3. The products were analyzed on 1% TAE agarose gels or loaded undiluted onto an Advanced Analytical Technologies Fragment Analyzer and analyzed according to the manufacturer’s instructions.

For GM14044, we pooled the product of 96 10 µL-reactions with 100 pg of genomic DNA as a template for each reaction. This was done to avoid amplification biases against longer alleles. We used MangoTaq as above but with the following PCR program: 95 °C for 60 s, then 25 cycles of 95 °C for 15 s, 55 °C for 15 s, and 72 °C for 270 s followed by 10 min at 72 °C. For sample GM03756A, we amplified 1 ng of genomic DNA as template using the MyFi Taq (Bioline). The PCR was done over 35 cycles using the same program as for GM14044 and using primers oVIN-1252 and oVIN-1320. The products were concentrated by alcohol precipitation.

Sanger sequencing was used to confirm all repeat sizes reported except for samples GM14044, NA05164, and GM03756A, which harbored repeat tracts too heterogeneous and too long to be sequenced. Note that this analysis also allowed us to determine the number of CCG repeats at the *HTT* locus (Supplementary Table S4). For the HD samples, we performed a small-pool PCR as described in^37^ (Supplementary Fig. S7). Briefly, the *HTT* repeat locus was amplified from genomic DNA with primers oVIN-1333 and oVIN-1334 for 30 cycles, with the first 5 cycles having an annealing temperature of 52 °C, raised to 55 °C for the following 25 cycles. The resulting products were run on a 2% agarose gel. The gel was then transferred on a charged nylon membrane by alkaline transfer and probed for the CAG repeats using [α-32P]-labeled oligo containing 10 CAG oligonucleotide repeats (oVIN-100). The membrane was exposed to a phosphoscreen and revealed using a Typhoon Scanner. The SP-PCR for the *DMPK* locus in sample NA05164 and GM03756A was done using primers oVIN-1251 and oVIN-1252 and the Ung-based protocol described previously^26^. This approach consists of treating the DNA with a heat labile Ung (Roche), which degrades any molecules containing uracil. The amplification is then done with dUTP instead of dTTP. This prevents carry-over contamination. The Taq polymerase used was Phusion U Green Hot Start (Thermo Fisher) along with 2 ng of genomic DNA per reaction.

To determine how closely sized alleles can be separated, we purified 4 alleles out of samples GM02168, NA13512, and GM14044. Their *HTT* locus was first amplified with oVIN-1333 and oVIN-1334 and the same PCR program as for GM14044. For each sample, we performed 10 PCR reactions, pooled the products, and loaded the resulting material on a 2% TAE agarose gel. Both alleles from GM02168 (15 and 30 CAGs) were gel-extracted together, and the bands with 19 and 46 CAGs amplified from GM14044 and NA13512, respectively, were gel-extracted individually. These purified bands were pooled at equimolar concentrations prior to on-chip analysis.

The unaltered pictures of DNA gels and SP-PCR blots can be found in Supplementary Fig. S9

### Chip fabrication and systems integration

The process flow for fabricating the microfluidic chip has been described in^8^. Briefly, the channels were obtained by plasma etching of silicon after photolithography. Two steps of etching were carried out over a depth of 16 µm away from the constriction to limit the hydraulic resistance of the microfluidic channel and 2 µm proximal to the constriction. The maximal and minimal channel widths were 800 and 5 µm, respectively. Inlets and outlets were then produced by sand blasting, and silicon was isolated with a layer of 300 nm of thermally-grown oxide. The devices were eventually sealed with a glass wafer by anodic bonding. Two chip designs have been used with linear and power-law shapes for the constriction (Supplementary Table S2); their comparison is discussed in^27^. Chips were inserted into a peek support and placed on a Zeiss microscope (see^38^ for details) equipped with a 10X objective (NA = 0.3) and an sCMOS camera with no binning (pixel size = 6.5 µm). A pressure controller MFCS (Fluigent) and an electric generator connected to the reservoirs by platinum electrodes were used for actuation. The chips were initially filled with ethanol then rinsed with buffer before loading the different DNA ladders and the samples. One chip could be used to process at least five samples.

Raw micrographs of each experiment presented in the manuscript can be found in Supplementary Fig. S10.

## Electronic supplementary material

Supplementary Material

Supplementary video
