# Supplementary material for: µLAS: Sizing of expanded trinucleotide repeats with femtomolar sensitivity in less than 5 minutes
Source: Sci Rep. 2019 Jan 10;9:23. doi: 10.1038/s41598-018-36632-5 (PMC6328573; doi:10.1038/s41598-018-36632-5)
Supplement: Supplementary file 1 — Supplementary Material [file 41598_2018_36632_MOESM1_ESM.docx]

**Supplementary Material**

**µLAS: Sizing of expanded trinucleotide repeats with femtomolar sensitivity in less than 5 minutes**

| Rémi Malbec^1^, Bayan Chami^1^, Lorène Aeschbach^2^ , Gustavo A. Ruiz Buendía^2^, Marius Socol^1^,Pierre Joseph^1^, Thierry Leïchlé^1^, Evgeniya Trofimenko^2*^, Aurélien Bancaud^1^, and Vincent Dion^2^    ^1^ LAAS-CNRS, Université de Toulouse, CNRS, Toulouse, France  ^2^ Center for Integrative Genomics, Faculty of Biology and Medicine, University of Lausanne, Bâtiment Génopode, 1015 Lausanne, Switzerland  * current address: Department of Physiology, University of Lausanne, Rue du Bugnon 7, 1005 Lausanne |
| --- |


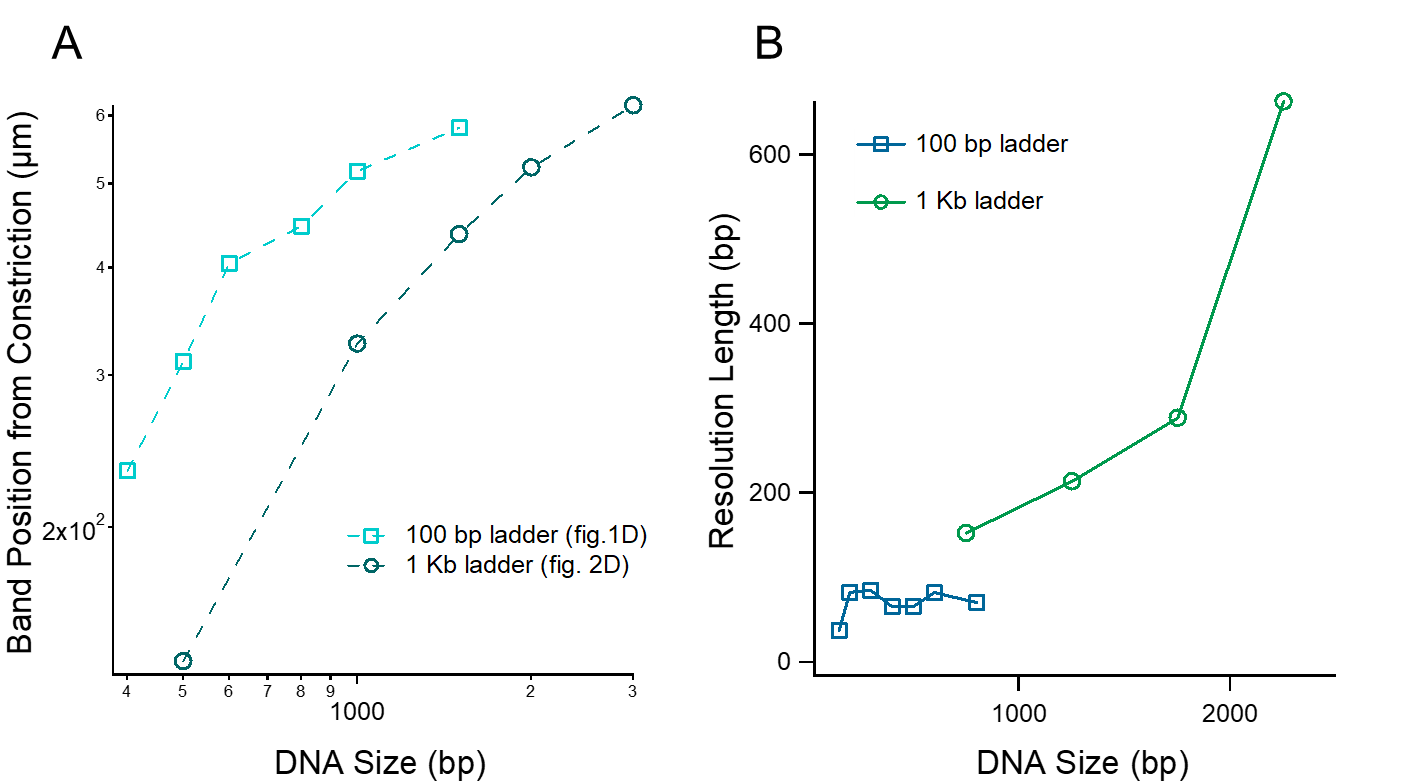


**Supplementary figure S1. DNA bands position and RSL as a function of their MW. (A)** The plot represents the position of DNA bands in the funnel region as a function of their size in bp extracted from figures 1D and 2D. The position vs. MW does not follow a homogeneous power-law scaling.  **(B)** The plot represents the resolution length (RSL) as a function of DNA size, as determined by measuring the resolution between the different bands of the two DNA ladders.


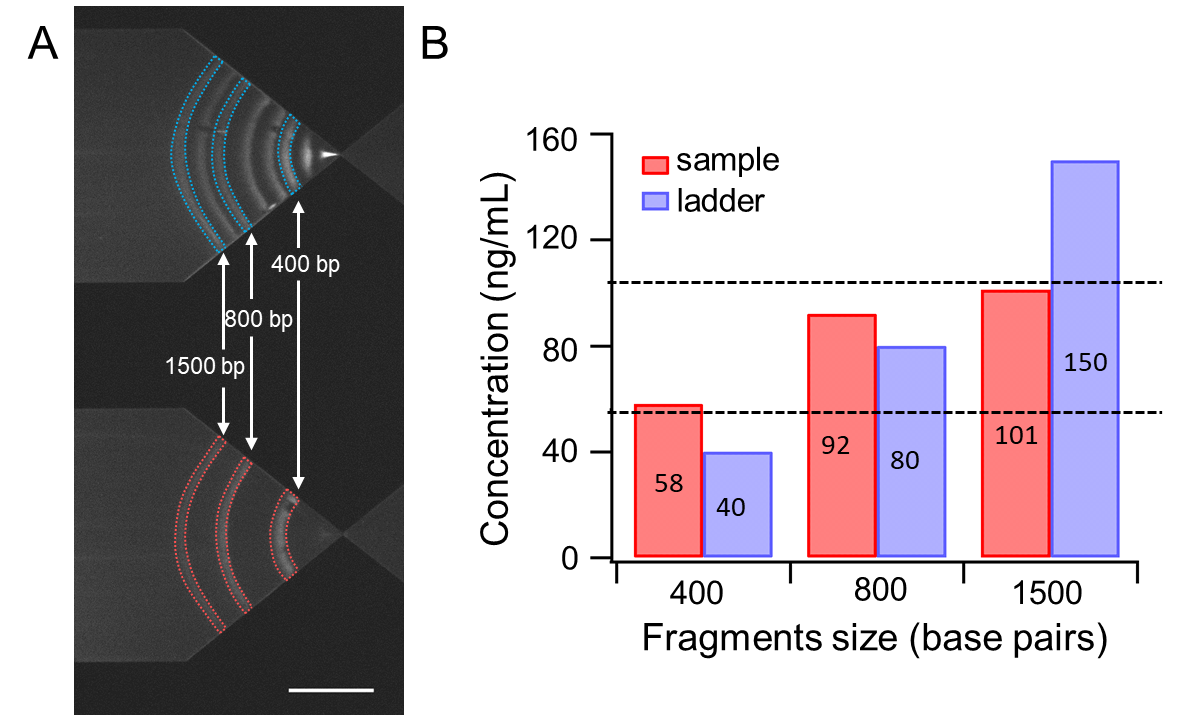


**Supplementary figure S2. Determination of band concentration by comparison of their intensity with respect to the ladder reference. (A)** The fluorescence micrograph shows the dual channel chip with the sample (bottom) and the ladder (top). The concentration of the three bands of the sample is 80 pg µL^−1^, and the bands of the ladder of 400, 800 and 1500 bp are diluted at concentrations of 40, 80 and 150 pg µL^−1^, respectively. The scale bar corresponds to 300 µm. **(B)** Fluorescence intensity is integrated in the 6 ROI of the image shown in (A) to estimate the amount of DNA in each band. Provided that we know the concentration of the reference bands in the ladder, fluorescence intensity ratio enable us to infer the concentration of the target bands of 58, 92, and 101 pg µL^−1^, as reported in the plot. The concentration is measured with an error of +/- 30% around the expected concentration of 80 pg µL^−1^


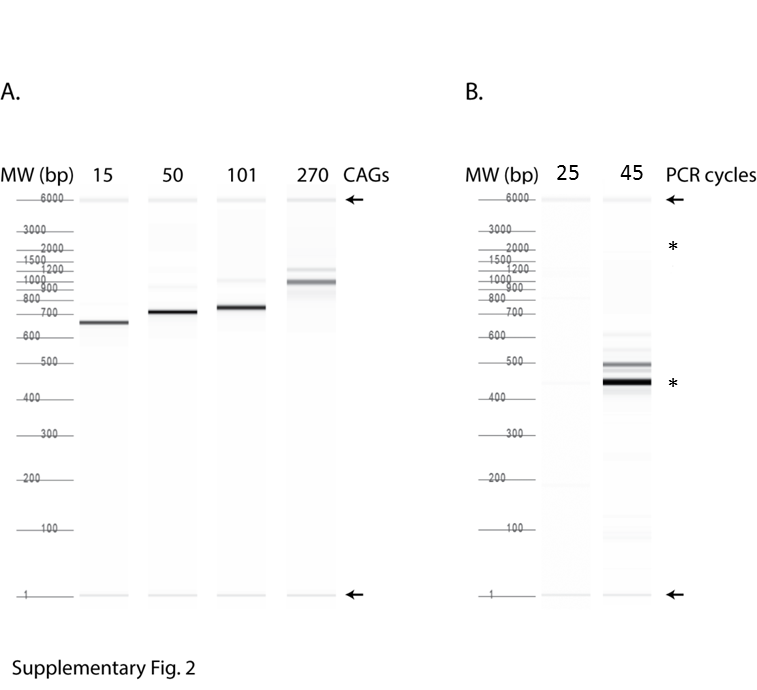


**Supplementary Fig. S3. PCR amplicons characterized with Fragment analyzer. (A)** Amplicons of a transgene with repeat sizes ranging from 15 to 270 CAGs in GFP(CAG)x previously characterized by Sanger sequencing (21). The minor bands are most likely the result of repeat instability, non-specific amplification, or the generation of heteroduplexes. **(B)** PCR amplicons of GM14044 after 25 and 45 cycles of PCR. Note that the sample at 45 cycles was diluted three times before being loaded onto the Fragment Analyzer. The stars represent the size of the expected PCR products. In both panels, the arrows are the spiked in length markers at 1 and 6,000 bp. On the 25^th^ cycle PCR, the Fragment Analyzer software detected a band at 443 bp and two peaks at 819 and 1119 bp.


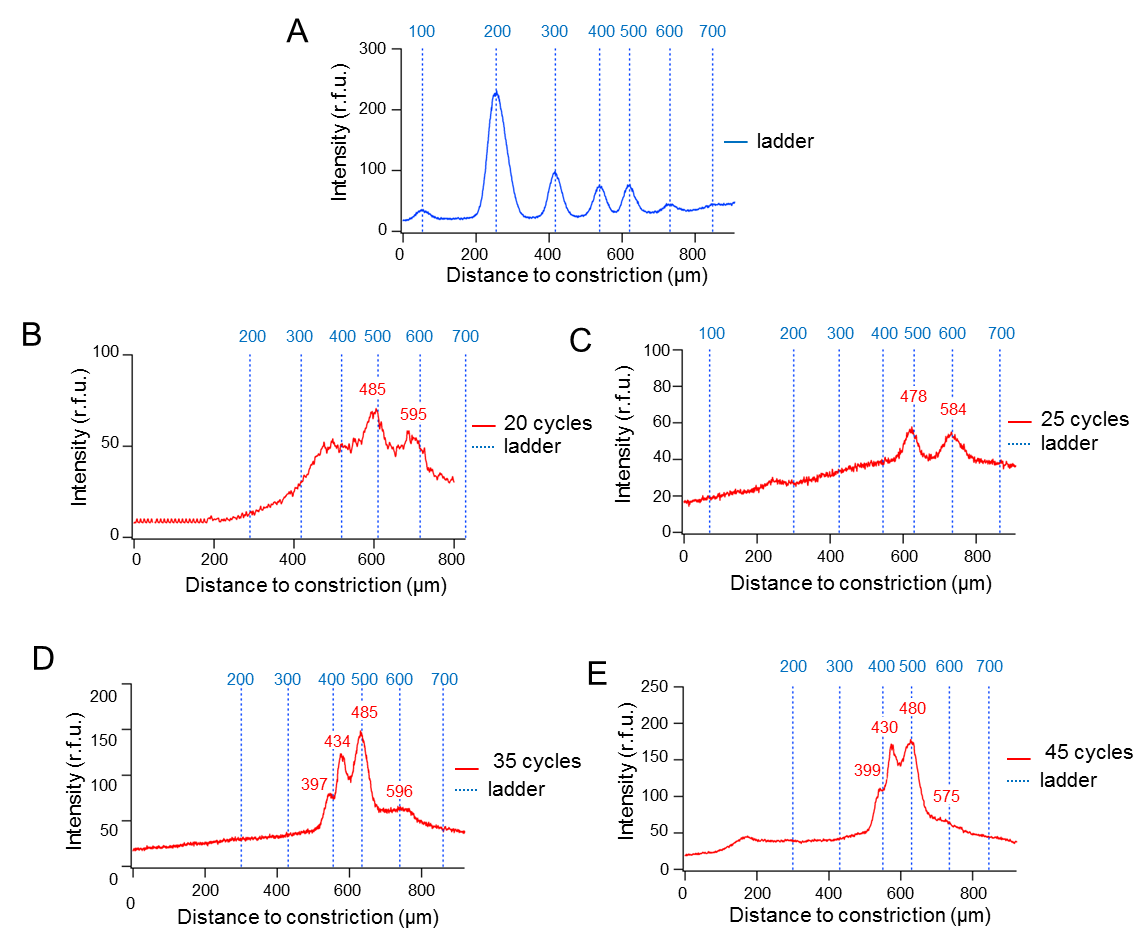


**Supplementary figure S4. Plots from the intensity distribution along the channel symmetry axis for sample GM03620 after different number of amplification cycles and corresponding to micrographs in Fig. 5.** Analysis of PCR products in the time course of the reaction. **(A)** Plot of the reference channel filled with ladder. **(B-E)** Sample after 20, 25, 35 and 45 cycles of PCR respectively.


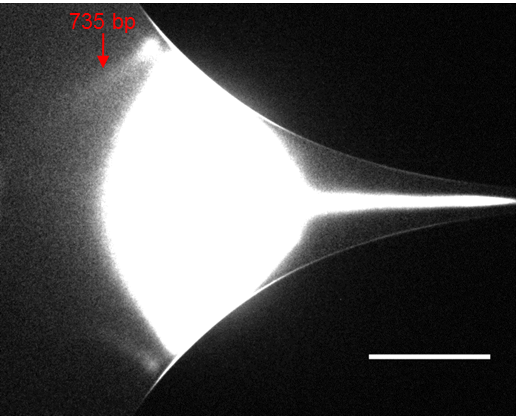


**Supplementary figure S5. Fluorescence micrograph of the sample GM03620 after 45 cycles of PCR using 100 s of µLAS enrichment at 2 bar and 130 V**. These actuation parameters correspond to ~5.7 cm s^−1^ and 1525 kV m^−1^ for the maximum flow velocity and electric field, respectively. The low intensity band in the left of the figure corresponds to a band of 735 bp. Note that the kinetics of concentration of high MW DNA bands is slower than that of low MW fragments, accounting for the bright signal ahead in the constriction (6). The scale bar corresponds to 200 µm.


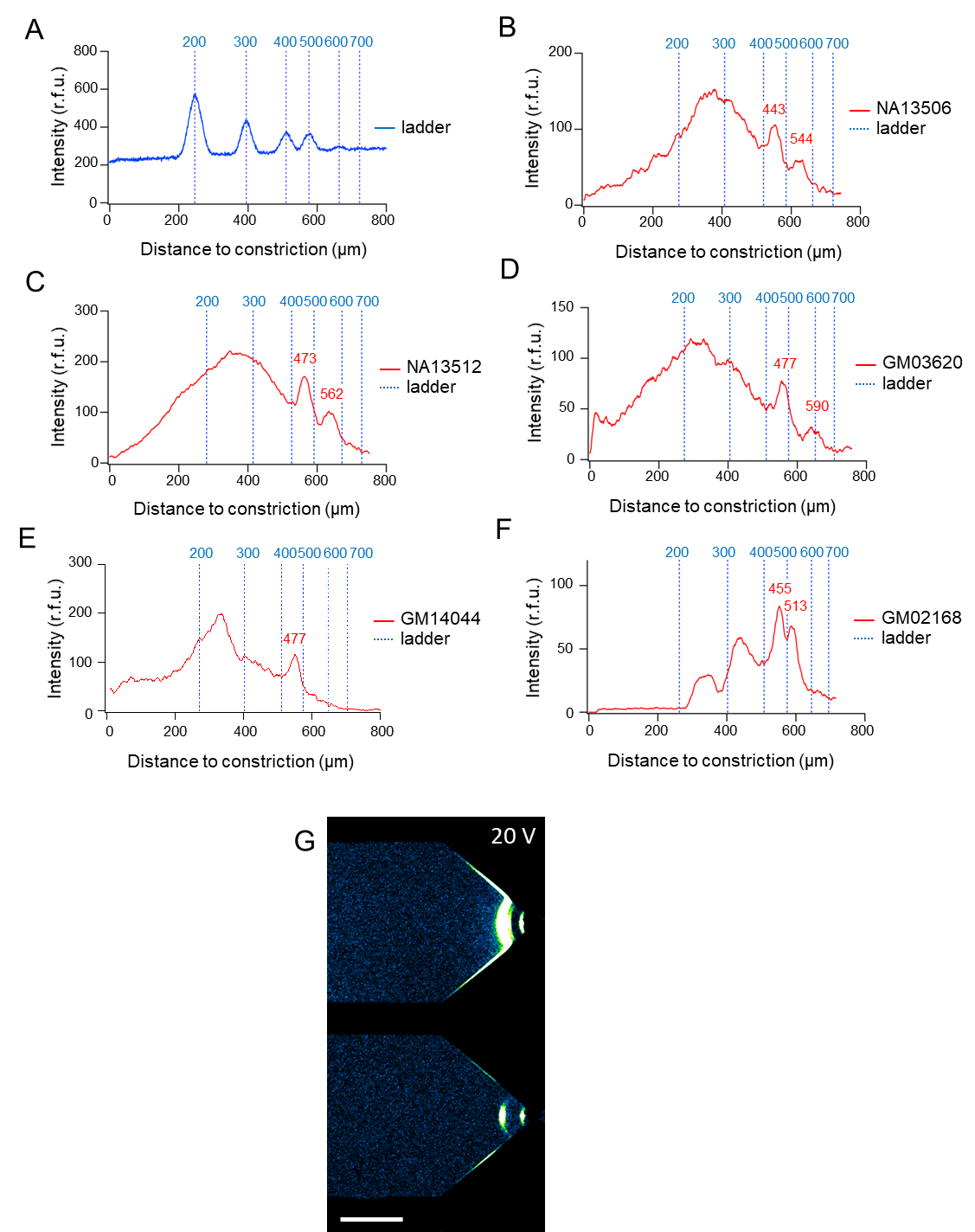


**Supplementary figure S6. Plots from the intensity distribution along the channel symmetry axis for different patient samples and corresponding to the micrographs in Fig. 6.** Analysis of patient samples after 25 cycles of PCR. **(A)** Plot of the reference channel filled with 100bp ladder. **(B-F)** Sample NA13506, NA13512, GM03620, GM14044 and GM02168 respectively. (G) The fluorescence micrograph represents the GM14044 PCR products conveyed in the chip at 1 bar and 20 V, *i.e.,* maximum hydrodynamic and electric fields of ~1.2 cm s^−1^ and 1700 kV m^−1^, respectively, and enriched during 30 s. The scale bar corresponds to 200 µm. The bands found below 400 bp are likely to be primers, which were not removed in these experiments.


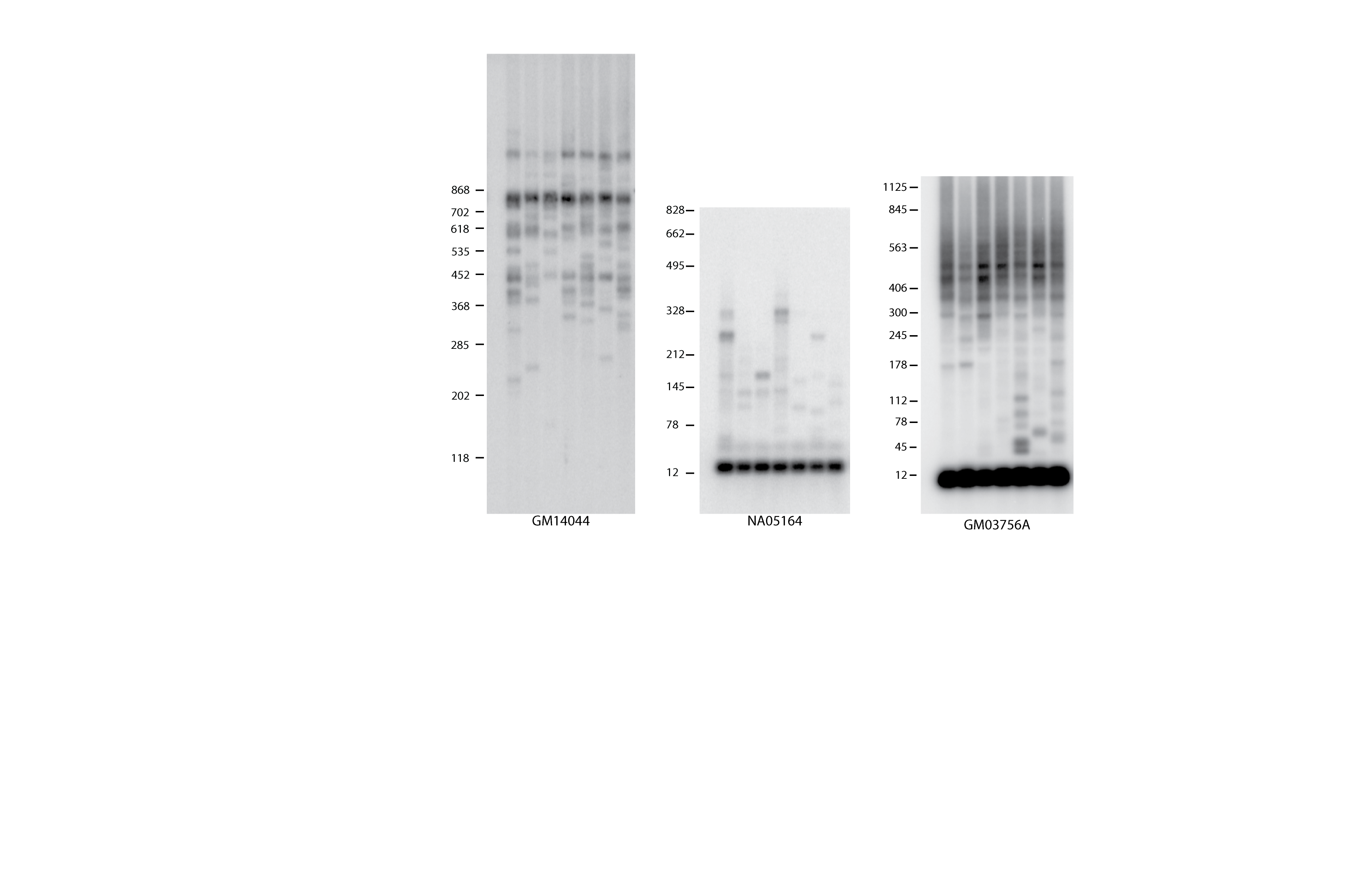


**Supplementary figure S7. SP-PCR analysis.** We confirmed the size of the expanded CAG repeats of samples GM14044 **(left)**, NA05164 **(middle)**, and GM03756A **(right)**, their average sizes, and the presence of extensive instability in these samples. The numbers on the left indicate the number of CAGs.


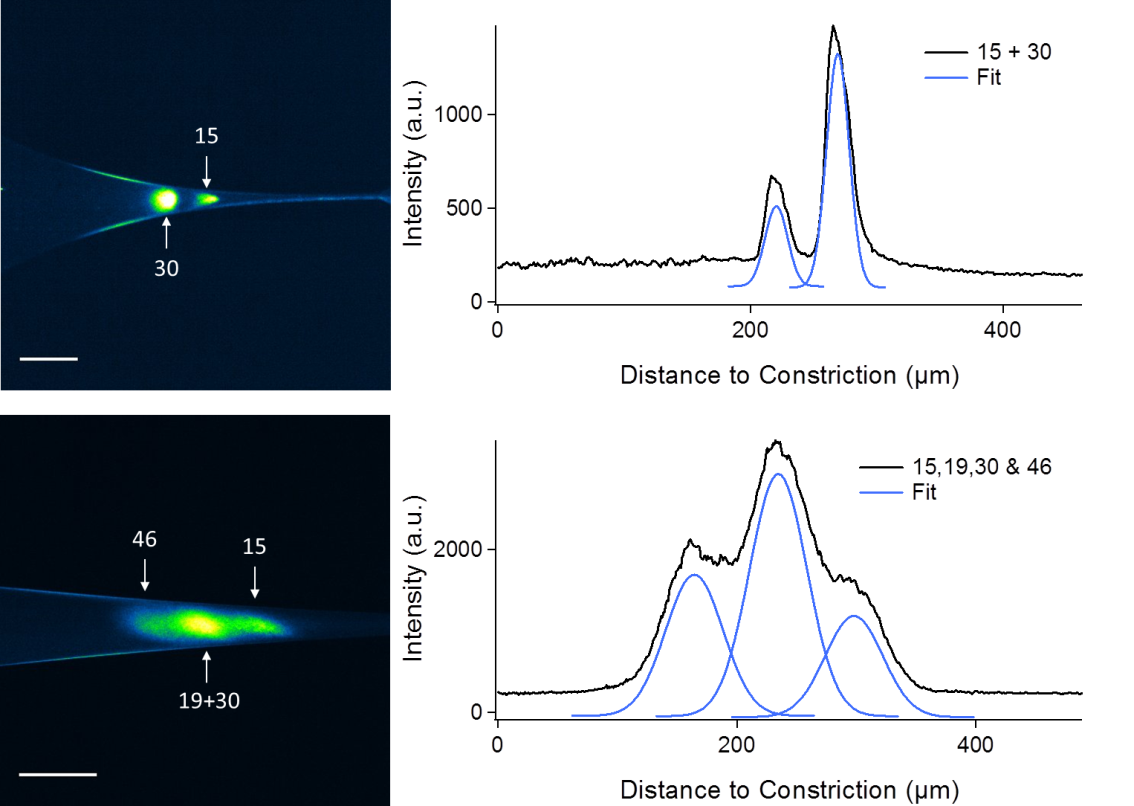


**Supplementary figure S8. Analysis of purified alleles containing 15, 19, 30 and 46 repeats in a single channel.** The upper panel shows the bands of 15 and 30 repeats that can be found in the patient sample GM02168. The sample is conveyed in the chip at 6 bar and 82 V, corresponding to maximal flow velocity and electric field of ~7.1 cm s^−1^ and 6.9 kV m^−1^, respectively, and enriched during 30 s. The image in the bottom panel is the same experiment with the four bands pooled together. The scale bar corresponds to 200 µm.


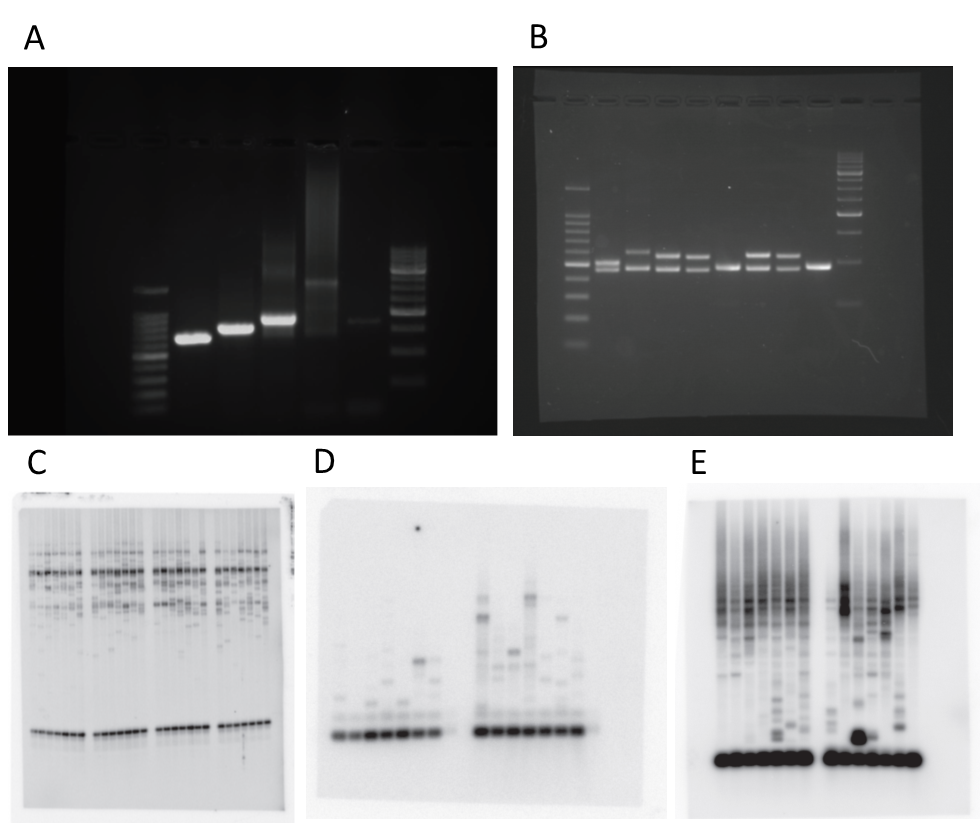


**Supplementary Fig. S9: Uncropped and unaltered gels**. (A) Gel from figure 3A. (B) Gel from figure 5A. (C-E) Gels from supplementary figure S1.
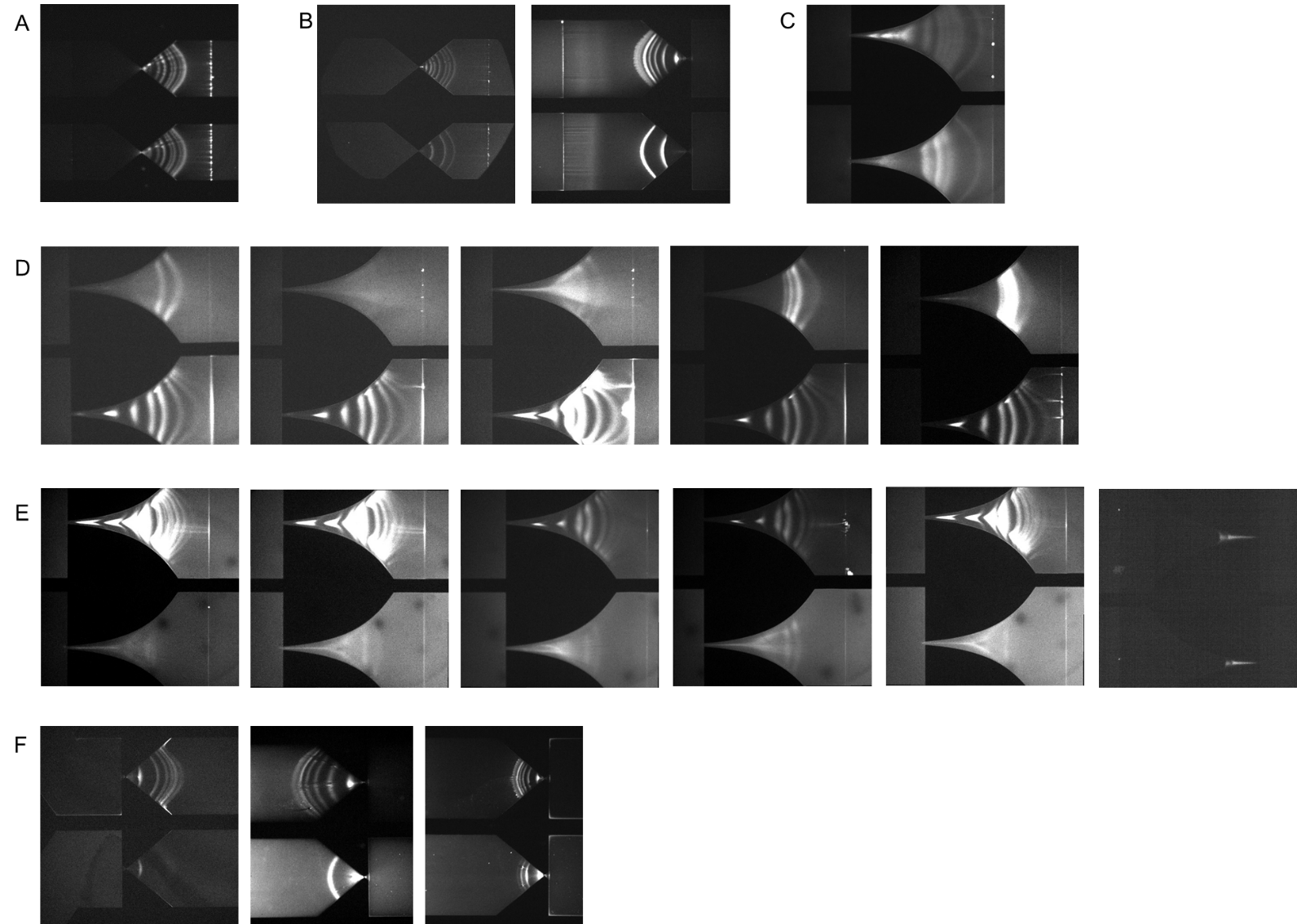


**Supplementary Fig. S10: Original micrographs from** µLAS**.** (A) Micrograph from figure 1A. (B) Micrographs from figure 2A (left) and 2D (right). (C) Micrograph from figure 4B. (D) Micrographs from figure 5. (E) Micrographs from figure 6. (F) Micrographs from figure 7.

**Supplementary table S1:** Reproducibility of µLAS. Separation and concentration were carried out four times and the positions of three peaks in a Kbp ladder (0.5 Kbp, 1.5 Kbp and 3 Kbp) and two sample peaks corresponding to 1091 bp and 3314 bp were systematically measured. For these five peaks, we determined the standard deviation (σ_pos_) for each measurement.

| **DNA band** | **Positon (µm)** | **σ_pos_** |
| --- | --- | --- |
| 500 bp  Upper channel | 143.6 | 2.00 |
|  | 145.2 |  |
|  | 145.9 |  |
|  | 141.3 |  |
| 1500 bp  Upper channel | 436.6 | 2.59 |
|  | 438.1 |  |
|  | 435.7 |  |
|  | 432 |  |
| 3000 bp  Upper channel | 582.2 | 4.04 |
|  | 574.9 |  |
|  | 572.9 |  |
|  | 575.5 |  |
| 1091 bp  Lower channel | 336.4 | 2.99 |
|  | 337.2 |  |
|  | 333.3 |  |
|  | 330.7 |  |
| 3314 bp  Lower channel | 610 | 3.18 |
|  | 603.3 |  |
|  | 610.1 |  |
|  | 607.3 |  |

**Supplementary table S2.** Expressions of the flow rate, flow velocity and electric field along the symmetry axis of the channel for the two designs used in the study. The comparison of these designs will be compared quantitatively in a manuscript in preparation. With *∆P* in bar, *l* in µm, *η* in cP and *V* in V.

| **Constriction Geometry** | 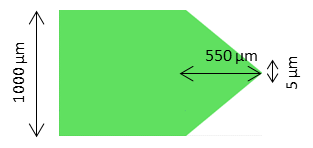 | 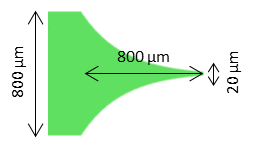 |
| --- | --- | --- |
| **Flow rate (µL/min)** | $Q=\frac{0.498 \Delta P\text{ }}{\eta}$ | $Q=\frac{3.26 \Delta P\text{ }}{\eta}$ |
| **Flow velocity at the center of the channel (m/s)** | $\text{V}\text{0}\text{(l)}=\frac{2.08 \Delta P}{\left( 1.59 l+5 \right)\eta}$ | $\text{V}\text{0}\text{(l)}=\frac{20.38 \Delta P}{\left( 40.46 {exp}^{0.00376 l}-20 \right)\eta}$ |
| **Electric velocity at the center of the channel (kV/m)** | $\text{E}\text{0}\text{(l)}=\frac{420 V}{\left( 1.59 l+5 \right)}$ | $\text{E}\text{0}\text{(l)}=\frac{240 V}{\left( 40.46 {exp}^{0.00376 l}-20 \right)}$ |

**Supplementary table S3.** Primers used in this study.

| Name | Sequence | Target | Expected size | Reference |
| --- | --- | --- | --- | --- |
| oVIN-0100 | 5’CAGCAGCAGCAGCAGCAGCAGCAGCAGCAG | Probe for SP-PCR | - | (29) |
| oVIN-1333 | 5‘CCGCTCAGGTTCTGCTTTTA | HTT | 401bp + (CAG)_n_ | This study |
| oVIN-1334 | 5‘CAGGCTGCAGGGTTACCG |  |  |  |
| oVIN-1425 | 5’GACCTCATACGAAGATAGGCTT | GFP insert in GFP(CAG)_X_ cell lines. | 627bp + (CAG)_n_ | This study and ref. (21) |
| oVIN-0460 | 5‘TCTGCAAATTCAGTGATGC |  |  |  |
| oVIN-0273 | 5‘ATT TTG CCG ATT TCG GCC TAT | pGEM-t-easy | 466bp | Primers: This study, plasmid: Promega |
| oVIN-0811 | 5‘TTTCTTGGCTTTATATATCTTGTGGAAAGGA  CGAAACACCGGATCATTGGAAAACGTTCTT |  |  |  |
| oVIN-0961 | 5‘CTGCAGGTCGACCATAGTGACT | pLenti CMV Puro DEST – 796bp fragment | 796bp | Primers: This study, plasmid:  Ref. (30) |
| oVIN-0597 | 5‘GCCTTGGGAAAAGCGCCTCC |  |  |  |
| oVIN-0124 | 5‘CGCCGCATACACTATTCTCA | pGEM-t-easy | 1536bp | This study |
| oVIN-0600 | 5‘GGCGCTCTTCCGCTTCCTCG |  |  |  |
| oVIN-1251 | 5’ GAGCGTGGGTCTCCGCCCAG | DMPK (SP-PCR) | 265 bp + (CTG)_n_ | This study |
| oVIN-1252 | 5’ CACTTTGCGAACCAACGATA |  |  |  |
| oVIN-1252 | 5’ CACTTTGCGAACCAACGATA | DMPK (analysis on chip) | 1036 bp + (CTG)_n_ | This study |
| oVIN-1320 | 5’ ATAACCTCCCCAACCTCGAT |  |  |  |

**Supplementary Table S4**. Using Sanger sequencing, we determined the size of the CGG repeats in the primers of the HTT alleles.

| Cell line | CAG repeat sizes | CCG repeat sizes |
| --- | --- | --- |
| NA13506 | 15/48 | 10/10 |
| NA13512 | 16/46 | 10/10 |
| GM03620 | 18/60 | 13/10 |
| GM14044 | 19/750 | 12/ND |
| GM02168 | 15/30 | 13/10 |

29. Dion,V., Lin,Y., Hubert,L., Waterland,R.A. and Wilson,J.H. (2008) Dnmt1 deficiency promotes CAG repeat expansion in the mouse germline. *Hum. Mol. Genet.*, **17**, 1306–1317.

30. Campeau,E., Ruhl,V.E., Rodier,F., Smith,C.L., Rahmberg,B.L., Fuss,J.O., Campisi,J., Yaswen,P., Cooper,P.K. and Kaufman,P.D. (2009) A Versatile Viral System for Expression and Depletion of Proteins in Mammalian Cells. *PLoS ONE*, **4**, e6529.
